# Changes in H3K27ac at Gene Regulatory Regions in Porcine Alveolar Macrophages Following LPS or PolyIC Exposure

**DOI:** 10.3389/fgene.2020.00817

**Published:** 2020-08-20

**Authors:** Juber Herrera-Uribe, Haibo Liu, Kristen A. Byrne, Zahra F. Bond, Crystal L. Loving, Christopher K. Tuggle

**Affiliations:** ^1^Department of Animal Science, Iowa State University, Ames, IA, United States; ^2^Food Safety and Enteric Pathogens Research Unit, National Animal Disease Center, USDA-Agriculture Research Service, Ames, IA, United States

**Keywords:** pig, alveolar macrophages, histone modifications, chromatin state, transcriptome, lipopolysaccharide, Poly(I:C)

## Abstract

Changes in chromatin structure, especially in histone modifications (HMs), linked with chromatin accessibility for transcription machinery, are considered to play significant roles in transcriptional regulation. Alveolar macrophages (AM) are important immune cells for protection against pulmonary pathogens, and must readily respond to bacteria and viruses that enter the airways. Mechanism(s) controlling AM innate response to different pathogen-associated molecular patterns (PAMPs) are not well defined in pigs. By combining RNA sequencing (RNA-seq) with chromatin immunoprecipitation and sequencing (ChIP-seq) for four histone marks (H3K4me3, H3K4me1, H3K27ac and H3K27me3), we established a chromatin state map for AM stimulated with two different PAMPs, lipopolysaccharide (LPS) and Poly(I:C), and investigated the potential effect of identified histone modifications on transcription factor binding motif (TFBM) prediction and RNA abundance changes in these AM. The integrative analysis suggests that the differential gene expression between non-stimulated and stimulated AM is significantly associated with changes in the H3K27ac level at active regulatory regions. Although global changes in chromatin states were minor after stimulation, we detected chromatin state changes for differentially expressed genes involved in the TLR4, TLR3 and RIG-I signaling pathways. We found that regions marked by H3K27ac genome-wide were enriched for TFBMs of TF that are involved in the inflammatory response. We further documented that TF whose expression was induced by these stimuli had TFBMs enriched within H3K27ac-marked regions whose chromatin state changed by these same stimuli. Given that the dramatic transcriptomic changes and minor chromatin state changes occurred in response to both stimuli, we conclude that regulatory elements (i.e. active promoters) that contain transcription factor binding motifs were already active/poised in AM for immediate inflammatory response to PAMPs. In summary, our data provides the first chromatin state map of porcine AM in response to bacterial and viral PAMPs, contributing to the Functional Annotation of Animal Genomes (FAANG) project, and demonstrates the role of HMs, especially H3K27ac, in regulating transcription in AM in response to LPS and Poly(I:C).

## Introduction

Innate immune responses have a fundamental role in protecting the host from infection (Riera Romo et al., 2016). Immune cells, such as monocytes/macrophages, dendritic cells, neutrophils, and other types of cells (i.e., epithelial cells), express pattern recognition receptors (PRRs) for the recognition of microbial components know as pathogen-associated molecular patterns (PAMPs). Upon activation, PRRs trigger intracellular signaling cascades to activate and/or modify expression of transcription factors that regulate immune related genes. Alveolar macrophages (AM) are the first sentinels of the respiratory tree and constitute the predominant immune cell in the steady state (Allard et al., 2018). AM are an important defense cell when viral or bacterial organisms invade deep into the lung to orchestrate the initiation and resolution of the immune response. AM maintain various effector functions, such as tissue repair, secretion of pro/anti-inflammatory proteins, phagocytosis, antigen presentation, and stimulation of mucus production (Joshi et al., 2018) as well as in the polarization of innate and adaptive immunity (Armstrong et al., 2019). Macrophage activation in response to PAMPs depends on the PRRs recognizing specific PAMPs, such as lipopolysaccharide (LPS) from Gram-negative bacteria by TLR4 or viral double-stranded RNA (dsRNA) by TLR3 or RIG-I, to activate signaling pathways leading to the induction of the immune transcriptional program (Escoubet-Lozach et al., 2011; Liu et al., 2012).

PRR activation and signaling also triggers epigenetic modulators that modify chromatin structure and as consequence, DNA accessibility (Lawrence et al., 2016; Zhang and Cao, 2019). Combinations of post transcriptional modifications (PTMs) such as histone modifications (HMs) at specific sites are commonly used to define the chromatin state and its influence on the transcriptional program. HMs can influence chromatin compaction and accessibility to the DNA, leading to suppression or enhancement of transcription by modulating the availability of gene promoter/enhancers to transcriptional machinery (Grabiec and Potempa, 2018). Although epigenetic patterns and transcriptional programs in response to the activation of RIG-I/MDA-5, TLR3 and TLR4 have been investigated in human and mice (Zhao et al., 2011; Saeed et al., 2014; Hoeksema and de Winther, 2016; Oh et al., 2018), the epigenetic regulation of gene expression during infection remains incompletely understood, especially in swine. Studies in swine have focused on the RNA response to LPS (Kapetanovic et al., 2013; Liu Q. et al., 2018) and Polyinosinic-polycytidylic acid [Poly (I:C)] (Loving et al., 2006; Chaung et al., 2010; Hu et al., 2016) but little has been reported on epigenetic control of the inflammatory response in pigs (Willems et al., 2015; Yang et al., 2016).

Although AM are the guardians of pulmonary homeostasis, AM can represent a permissive niche for intracellular pathogens (Allard et al., 2018). Respiratory diseases in human and animals have captured the attention of researchers in the last years. In pigs, respiratory diseases are caused by several pathogens such as porcine reproductive and respiratory syndrome virus (PRRSV), porcine circovirus type 2 (PCV2), swine influenza A virus (IAV), and *Mycoplasma hyopneumoniae* (Mhp) resulting in significant economic losses in the swine industry worldwide and, for influenza, increasing the risk of zoonotic disease spread (Opriessnig et al., 2011; Wang et al., 2017; Anderson et al., 2020). There is strong evidence that the pig is a relevant model for human infectious diseases (Mair et al., 2014; Parnell and Volk, 2019). For example, the pig is more similar to the human than other models in terms of anatomy, physiology, pathophysiology, and phylogenetics (Meurens et al., 2012), and pig immune genes are more closely related at the DNA sequence level to humans than are mouse genes (Dawson et al., 2013).

To contribute to functional annotation of the porcine genome (part of the FAANG project), as well as better understand and utilize the pig as a human disease model, a comprehensive genome-wide annotation of HMs to construct a chromatin state map of the immune response is needed (Giuffra et al., 2019). In this study, we aimed to establish a chromatin state map of AM and determine the role of PTMs on the AM transcriptional response following stimulation with bacterial and viral mimics. We first used RNA-seq to determine the gene expression of AM stimulated with LPS or Poly (I:C), and performed ChIP-seq for 4 specific HMs to characterize the chromatin state under these treatments. Next, we performed an integrated bioinformatics analyses of the ChIP-seq and RNA-seq data to determine the functional regulatory role of chromatin modifications on gene expression. Overall, we describe a well-defined distribution of four histone marks, which correlated with differential gene expression of nearby immune related genes and their associated pathways. The analysis further demonstrated a stimuli-specific association of the H3K27ac mark with inflammatory response to LPS and Poly (I:C). Taken together, this first integrated analysis of HMs in porcine AM in response to both bacterial and viral mimics demonstrates the critical role of epigenetic signals in the innate immune response.

## Materials and Methods

### Animals

Cells were isolated from eight crossbred (predominantly Large White and Landrace heritage) pigs approximately 8–13 weeks of age. Eight pigs (four males and four females) were housed in BLS2 rooms at the National Animal Disease Center (Ames, IA, United States) and all animal procedures were performed in compliance and approval by Institutional Animal Care and Use Committee.

### AM Isolation, Culture and Treatments

After necropsy, lungs with trachea attached were removed from the body for collection of alveolar macrophages as previously described (Loving et al., 2006). Lungs were lavaged with 300 mL of 1x PBS supplemented with 100 μg/mL of gentamicin and ∼200 mL was recovered. Cells in collected lavage fluid were pelleted by centrifuged at 530 *g* for 10 min at 4°C. Cells were resuspended in supplemented medium (RPMI 1640, 5% swine sera, 25 mM HEPES, 2 mM L-glutamine, 1% penicillin-streptomycin and gentamicin 100 μg/mL (Invitrogen life technologies) and seeded into 150 × 15 mm petri dishes for overnight incubation at 39°C and 5% CO_2_. Non-adherent cells were then removed by gently pipetting and discarding the media. Adherent AM were released with a cell scraper, collected and washed once. Cell count and viability data were obtained using the MUSE cell analyzer system (Millipore). AM from each of the eight pigs were aliquoted and seeded in six 100 x 15 mm petri dishes with a final volume of 5 mL and rested for 2 h. AM culture in each petri dish were stimulated for 2 or 6 h at 39°C and 5% CO_2_ with 0.5 μg/mL Poly (I:C) (LMW) / LyoVec^™^ (InvivoGen) or 1 μg/mL LPS from *Escherichia coli* O55:B5 (Sigma). Non-stimulated cells were included at each time point as controls. At indicated times after stimulation, AM were gently scraped and collected to determine number and cell viability for subsequent molecular assays. For chromatin immunoprecipitation, aliquots of AM from each animal were fixed for 10 min by adding 16% paraformaldehyde (Electron Microscopy Science) to a final concentration of 1%. Fixation was stopped using 2.5 M glycine (Boston Bioproducts) to a final concentration of 150 mM, and cells were thoroughly washed in cold PBS. Cell pellets were snap-frozen in liquid nitrogen and stored at −80°C. For RNA work, cell aliquots were stored in RLT buffer and stored at −80°C. A flowchart of the methods is shown in the Supplementary Figure S1.

### RNA Isolation and Real Time PCR (qPCR)

RNA extraction from 1,000,000 cells was performed using the RNeasy Mini Kit (QIAGEN) following manufacturer’s instructions. Eluted RNA was treated with DNase Max Kit (QIAGEN) to break down and remove traces of DNA. RNA quantity and integrity were assessed with the Agilent 2200 TapeStation system (Agilent Technologies) and only samples with high RNA integrity numbers (RIN ≥ 7.6) were used for further analysis. For qPCR, a panel of five selected genes (*IL6*, *IL8*, *IL1β*, *TNF*, and *CASP1*) involved in inflammatory response were assayed to confirm the LPS and Poly (I:C) stimulation in AM from all animals. cDNA was synthesized using IScript^™^ Reverse Transcription Supermix from 400 ng of RNA according to manufacturer’s recommendation (BIO-RAD). The final 10 μl PCR reaction included 1 μl of 1:3 diluted cDNA as template, 5 μl of iTaq Universal SYBR^®^ Green Supermix (Bio-Rad), and gene specific forward and reverse primers at 20 μM to final concentration of 1 μM. QPCR was performed on a QuantStudio 5 system (Applied Biosystems) under following conditions: 95°C for 30 s; 95°C for 15 s followed by 60°C for 30 s (40 cycles), and a final dissociation step. Melting curve analysis was performed, to check specificity of each PCR product. Levels of mRNA were calculated according to the 2 ^–ΔΔ*CT*^ method (Livak and Schmittgen, 2001), which represent mRNA abundance in stimulated AM relative to non-stimulated AM after normalizing to *HPRT1* (Nygard et al., 2007). Final results were log_2_ transformed and statistical differences in expression levels between stimulated and non-stimulated were assessed using Kruskal-Wallis-one-way ANOVA and multiple comparison adjustment was performed (Graphpad Prism 6). Tests with *P*-value < 0.05 were considered statistically significant. Primers sequences are listed in Supplementary Table S1. Additionally, a Pearson correlation analysis was performed to validate RNA-seq data by qPCR. The significance level was set at *P* < 0.05.

### Stranded RNA-Sequencing

RNA from non-stimulated and stimulated AMs at 2 and 6 h from all animals (*n* = 4, two males and two females) were used for library preparation. The RNA was fragmented and prepared into sequencing libraries using the TruSeq Stranded Total RNA Sample Preparation Kit (Illumina). The 24 libraries were diluted and pooled together in approximately equimolar amounts. This pool of libraries was sequenced on an Illumina HiSeq 3000 sequencer in a 100 cycle, paired-end mode across three lanes.

### RNA-Seq Data Processing

Quality of raw reads were checked using FASTQC (version 0.11.7)^1^. Illumina sequencing adapters and low-quality bases were trimmed using the software Trimmomatic (version 0.36) (Bolger et al., 2014) with the following explicit settings: ILLUMINACLIP:adapters.fa:2:30:7:1:true LEADING:3 TRAILING:3 SLIDINGWINDOW:4:10 MINLEN:25, and A-rich stretches at 3′ termini and T-rich stretches at 5′ termini of length great than 10 bases were trimmed using custom Perl scripts if the contents of A or T were greater than 70%, respectively. After trimming, reads longer than 25 bases were re-paired using BBMap (version 38.16)^2^. For mapping, trimmed single end and paired-end reads were separately mapped to the reference genome *Sus scrofa* 11.1 (Ensembl, version 90) using the aligner STAR (version 2.5.3a) (Dobin et al., 2013) with the following explicit settings: –runThreadN 8 –readFilesCommand zcat –outFilterType BySJout –outFilterMultimapNmax 20 –alignSJoverhangMin 8 –outFilterIntronMotifs RemoveNoncanonical –alignSJDBoverhangMin 1 –outFilterMismatchNmax 30 –seedSearchStartLmax 50 –seedSearchStartLmaxOverLread 0.5 –alignIntronMin 20 –alignIntronMax 1000000 –alignMatesGapMax 1000000 –outSAMstrandField intronMotif –outSAMtype BAM, and the GTF file (Ensembl, version 90) as input for known splicing junctions. A second round of mapping was performed with the same explicit settings except the novel splicing junctions detected in the first round were used as input for another option: –sjdbFileChrStartEnd. Gene-level read counts resulting from mapping based on paired-end and single end reads were determined separately using featureCounts (version 1.6.0) (Liao et al., 2014) with the following explicit settings: -d 25 -Q 255 -s 1 -T 8 –primary, and along with the GTF file (Ensembl, version 90) as the genome annotation file and summed up for each gene of each sample. The resulting count table was filtered to remove genes with extremely low expression levels such that only genes with > 1 count per million reads mapped (CPM) in at least four samples were kept. Thus, the final count table contained 11,091 genes across the 24 samples, which was used for differential gene expression analysis.

### Differential Gene Expression Analysis

Differential gene expression analysis was performed using the R/Bioconductor package DESeq2 (version 1.24.0) (Love et al., 2014). To account for the hidden variations introduced by the RNA-seq, surrogate variable analysis (SVA) was applied. Four surrogate variables were determined using the svaseq function in the sva package (Leek, 2014) with a full model containing pig individuals and the combinations of treatment and time points as independent variables, and a reduced model with pig individuals as the only independent variable.

A generalized linear model was fitted for each gene in the count table, with a negative binomial response and a log link that included a DEseq2 normalization offset and the effects of pig individuals, time, treatment and time-by-treatment interaction, and the four surrogate variables as estimated above. The *nbinomWaldTest* function was used to estimate and test the significance of regression coefficients with the following explicit parameter settings: betaPrior = FALSE, maxit = 5000, useOptim = TRUE, useT = FALSE, useQR = TRUE. Differentially expressed genes between conditions were identified by testing the significance of relevant contrasts and using the *results* function with the following explicit parameters: alpha = 0.01, lfcThreshold = log_2_(1.5), altHypothesis = “greaterAbs”. Nominal p values were adjusted for multiple testing using the BH method (Benjamini and Hochberg, 1995). Genes with | log_2_(fold change) | > log_2_(1.5) and adjusted *p*-value < 0.05 were considered to be differentially expressed genes (DEGs).

Additionally, a Spearman correlation analysis was performed to validate gene expression and a Wilcoxon rank-sum test was used validate the gene expression similarity across species. The significant level was set at *P* < 0.05 for Spearman test and *P* < 0.05 for Wilcoxon test (since the null hypothesis is that the gene lists were not different).

### Gene Ontology (GO) Enrichment Analysis

Functional enrichment analysis based on a hypergeometric distribution was performed using ClueGO (Bindea et al., 2009), a Cytoscape plug-in. The GO terms and Kyoto Encyclopedia of Genes and Genomes (KEGG) pathways were ranked using the significance term. The criteria for statistically significant enrichment were set as follows: BH-adjusted *p*-value < 0.05, κ score > 0.5 and at least 3 genes per term. All Ensembl Gene IDs with detectable expression level in alveolar macrophages (*n* = 11,091) were used as the background reference and the gene ontology annotation^3^ for *Sus scrofa* was used as reference.

### Chromatin Immunoprecipitation Followed by Deep Sequencing (ChIP-Seq) and Data Analysis

The AM used for ChIP-seq experiment were from the same four pigs used in the RNA-seq experiment. H3K4me3 (active promoter regions) and H3K27me3 (associated with Polycomb repression) were performed using AM from two biological samples (1 male and 1 female), and the other two histone marks H3K27ac (enhancer and promoter regions) and H3K4me1 (enhancer and promoter regions) were performed on the other two biological samples (1 male and 1 female). While on ice, pellets of 200,000 fixed cells (for H3K4me3 and H3K27me3) or 400,000 fixed cells (for H3K4me1 and H3K27ac) were resuspended in 1 mL of cell lysis buffer (50 mM Tris pH 8.0, 140 mM NaCl, 1 mM EDTA, 10% glycerol, 0.5% NP-40 and 0.25% Triton X-100) supplemented with Pierce EDTA-Free protease inhibitor mini tablets (ThermoFisher Scientific). Eosin staining was used to determine the viability and number of nuclei in a hemocytometer. The nuclei pellet was resuspended in 140 μL of nuclear lysis buffer (10 mM Tris pH 8.0, 1 mM EDTA, 0.5 mM EGTA and 0.5% SDS) supplemented with Pierce protease inhibitor tablets (ThermoFisher Scientific), and chromatin was sheared using a focused ultrasonicator (Covaris ME220) with the following parameters: target size 350 bp, 90 seconds of shearing, 70 peak power, 20% duty factor, 1000 cycles per burst. ChIP was performed using iDeal ChIP-seq kit for histones (Diagenode) following manufacturer’s protocol. The following amounts of antibodies from Diagenode were used for ChIP-seq: 1.5 μg H3K4me3 (C15410195), 1 μg H3K4me1 (C15410194), 2 μg H3K27ac (C15410196) and 1 μg H3K27me3 (C15410003). Sequencing libraries for H3K4me3 and H3K27me3 ChIP and the corresponding input samples were prepared using the NEBNext Ultra II DNA library Prep Kit (Illumina). For H3K27ac and H3K4me1 ChIP and the corresponding input samples, the ACCEL-NGS^®^ 2S Plus DNA Library kit was used, following the manufacturer’s instructions. The libraries were pooled together and sequenced on an Illumina HiSeq 3000 sequencer to generate 100-bp paired-end reads across five lanes.

### ChIP-Seq Data Processing

Read quality was checked using FASTQC (version 0.11.7)^4^. Illumina sequencing adapters and low-quality bases were trimmed using Trimmomatic (version 0.36) (Bolger et al., 2014) with the same setting used for RNA-seq data trimming. Paired-end, trimmed reads were separately mapped to the pig reference genome Sus scrofa 11.1 using the aligner BWA mem (Li and Durbin, 2009) with the explicit settings: -M -t 8. The alignment output in the SAM format was converted into BAM, followed by sorting and indexing, using SAMtools (Li et al., 2009). Duplicates in BAM files of paired-end and single end reads were marked separately, and then BAM files for the same samples were merged. Library insert size and duplication rate were checked using picard tools^5^. The deeptools (Ramírez et al., 2014) and the ChIPQC package (Carroll et al., 2014) were used for ChIP-seq data quality control.

### Genetic Similarity Analysis

Reads from whole genome sequencing data for the four input samples of the ChIP-seq experiment were aligned to the pig reference genome and duplicates were marked as described above. Genetic variants were called using the software GATK (version 3.8) (Poplin et al., 2018) by following the best practices for germline cohort joint short variant discovery^6^. SNP variants were filtered using the following settings: “QD < 2.0 || FS > 60.0 || MQ < 40.0 || MQRankSum < −12.5 || ReadPosRankSum < −8.0 || SOR > 3.0”. The genetic similarity scores between the four pigs were calculated using the snpgdsIBS function of the SNPRelate package (version 1.16.0) (Zheng et al., 2012), with 12,949,995 SNPs (autosomal, non-monomorphic, and missing genotype-free) as input.

### Chromatin State Inference

Genome-wide chromatin states for each sample were predicted using the software ChromHMM (Ernst and Kellis, 2017). Given the high reproducibility of the ChIP-seq signal between biological replicates, ChIP-seq data for HMs H3K4me3 and H3K27me3 (Pigs 10 and 11) were assumed to be from the other two pigs (Pigs 7 and 8), from which ChIP-seq data for HMs H3K4me1 and H3K27ac were obtained. By referring to the publication of the RoadMap consortium (Kundaje et al., 2015), where 15 chromatin states were predicted based on ChIP-seq data for five histone marks, we performed chromatin state prediction by assuming 10 states. The Integrative Genomics Viewer (IGV) was used to visualize the chromatin states and the genome-wide distributions of histone marks.

### Identification of Histone Modification Enriched Regions (HMERs) and Differential Histone Modification Regions (DHMRs)

Punctate (H3K4m3, H3K4me, and H3K27ac) and broad (H3K27me3) histone modification enriched regions (HMERs) were identified using the software MUSIC (Harmanci et al., 2014) with the pre-processed BAM files as input. Irreproducible discovery rates (IDR) between genomic regions with enriched histone modification of every two biological replicates were calculated using IDR (Li et al., 2011). Differential histone modification regions were identified by using the software diffReps (Shen et al., 2013) using the common settings: –meth nb –pval 0.0001 –nrpass 1 –frag 200 –nproc 8 –noanno –nohs, and histone mark-dependent settings: –mode block –window 10000 –step 1000 –nsd broad for H3K27me3 ChIP-seq data, or –mode peak –window 1000 –step 100 –nsd sharp for ChIP-seq data of the other three histone marks. Genomic regions with histone modification level differing by two folds and false discovery rate (FDR) < 0.05 were considered as differential HMERs (DHMRs). HMERs and DHMRs were associated to the nearest transcripts using the package ChIPSeeker (Yu et al., 2015). Genome-wide distribution of HMERs and DHMRs among different genomic features were also determined using the same package ChIPSeeker (Yu et al., 2015).

### Principal Component Analysis of Histone Modification Profile

Histone modification enriched regions identified by MUSIC were merged into a super union set of enriched regions using the DiffBind package (version 2.10.0)^7^ with the minOverlap parameter set to 1. The RPKM fold enrichment, that is, log_2_ (RPKM_*ChIP*_/RPKM_*Input*_), were calculated for each genomic regions of the super union set for each sample using the dba.count function of the DiffBind package and used for principal component analysis using the princomp function in the stats (version 3.6.2) package^8^. A two-dimension PCA plot showing the relationship of the ChIP-seq samples were generated using the autoplot function of the ggfortify package^9^.

### Integration of ChIP-Seq and RNA-Seq Data

Expression level of each transcript was determined using the quasi-mapping tool Salmon (version 0.9.1) (Patro et al., 2017) based on the RNA-seq data. Transcripts were assigned to one of four bins based on their expression levels: high (> 9.5 TPM), medium (1.7 ∼ 9.5 TPM), low (0.28 ∼ 1.7 TPM), or no expression (< 0.28 TPM) bins. Deeptools was used to calculate and generate density plots for aggregated median read coverage normalized against the corresponding ChIP input read coverage for transcription start sites (TSS) ± 5 kb regions of transcripts in each bin.

Differentially expressed genes with DHMRs were identified from the intersection of the list of genes with assigned DHMRs and the list of DEGs for each compared condition. Hypergeometric tests were performed to check the significance of overlapping between the two lists of interest.

### Transcription Factor Motif Analysis

Histone modification-enriched genomic regions of biological replicates under each condition were separately merged using bedtools (version 2.29.2) (Quinlan and Hall, 2010). Known motifs of vertebrate transcription factors built in the software HOMER (version 4.11.1) (Heinz et al., 2010) were identified among merged genomic regions enriched for given histone modifications under given conditions using the following settings: “-size given -mask -mset vertebrates”. Random genomic regions with GC-content matching the input genomic regions were used as background for motif analysis by HOMER. Metascape analysis (Zhou et al., 2019) was performed for GO analysis of the transcription factors that bind the predicted motif. The threshold P-value was set to 0.01. Several terms were clustered into the most enriched GO term. Term pairs with Kappa similarity score above 0.3 were displayed as a network to show relationship among enriched terms. Terms associated with more genes tend to have more significant *P*-values. The protein-protein interactions were extracted from STRING (Han et al., 2004) and the Molecular Complex Detection (MCODE) (Bader and Hogue, 2003) algorithm was used in order to identify neighborhood components with a particular function. All networks displayed were visualized using Cytoscape.

## Results

### Inflammatory Response to LPS and Poly (I:C) in Porcine Alveolar Macrophages (AM)

**FIGURE 1:**
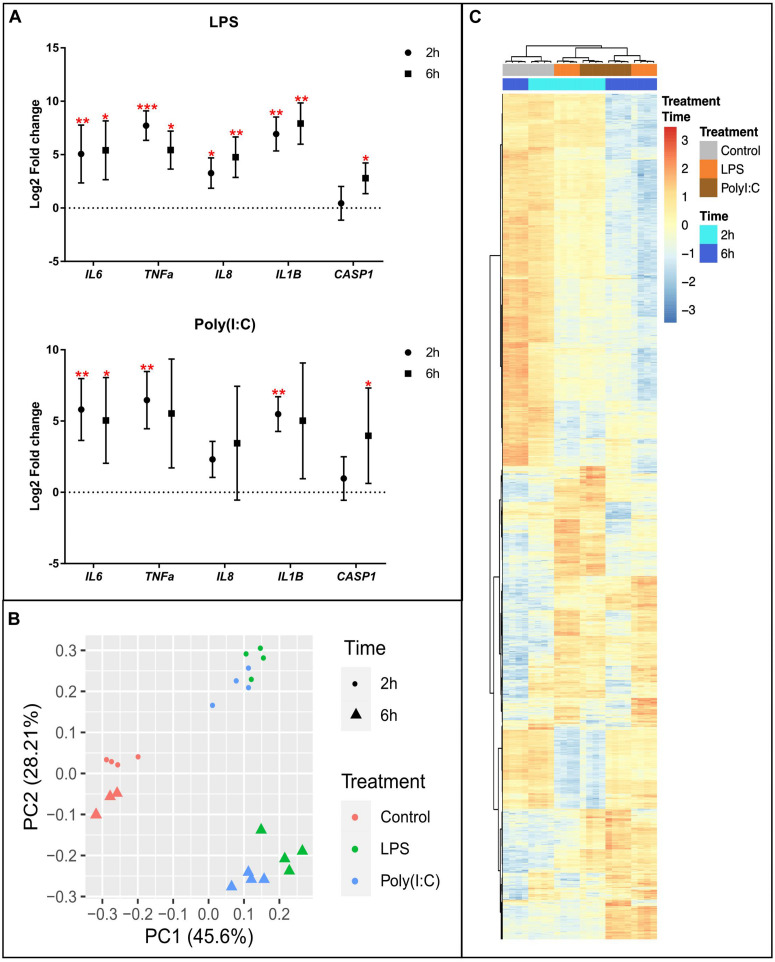
Transcriptional response of alveolar macrophages to LPS or Poly (I:C). **(A)** QPCR results of inflammatory marker genes in AM stimulated with LPS and Poly (I:C). Data are shown as the mean of Log2 Fold change +/–SD (*n* = 8). Kruskal-Wallis-one-way ANOVA was used to compare treatments with non-stimulated AM. Significance was set at *P* < 0.05. *P < 0.05, **P < 0.01, ***P < 0.001, ****P < 0.0001, see Supplementary Table S2 for details. **(B)** Principal component analysis of transformed RNA-seq reads counts for whole transcriptomes. Axis indicate component scores **(C)** Heatmap showing DEG between non-stimulated and LPS or Poly(I:C) stimulated AM at 2 and 6 h. Color code is based on Z-score of log_2_ transformed CPM across all samples. Genes, treatments and timepoints were hierarchically clustered (row, genes; columns, treatments and timepoints).

AM from eight healthy pigs were isolated by lavage, cultured overnight, and subjected to stimulation for 2 or 6 h (h) by TLR4 (LPS) or TLR3 and RIG-I/MDA-5 (Poly (I:C) agonists or media-alone as control, and collected for multiple analyses. To confirm the induction of an inflammatory response, we isolated total RNA and performed RT-qPCR to quantify differential expression of genes involved in inflammatory response. An increase in RNA levels of *IL6*, *IL1B*, *TNF*, *CASP1* and *IL8* were confirmed for both stimulations at each timepoint (Figure 1A and Supplementary Table S2).

### LPS and Poly (I:C) Induced Large-Scale RNA Response Across Multiple Pathways in Porcine AM

To investigate global induction of the RNA response in AM stimulated with LPS and Poly (I:C), we performed genome-wide expression analysis using RNA-seq. An average of 45,330,687 clean reads were produced from three independent treatments [culture media, LPS and Poly (I:C)] and two timepoints, each with four biological replicates (Supplementary File S1). Clean sequence reads were mapped to the *Sus scrofa* genome version 11.1, and an average of 41,029,762 (90.50%) were uniquely mapped. We used Principal Component Analysis (PCA) to obtain an overview of the similarities and differences between treatments as well as the time effect of culture. The first principal component (PC)-1 explained the variance and clearly separated stimulated from non-stimulated AM (Figure 1B) demonstrating that expression pattern changes due to both treatments were different from non-stimulated AM and more similar to each other. The second PC separated the samples by the time effect of the treatments, and also showed that LPS and Poly (I:C) treatment induced similar transcriptional profiles at 2 and 6h. All the transcriptional profiles were further confirmed by hierarchical clustering analysis (HCA) (Supplementary Figure S2). PCA and HCA based on gene expression data adjusted for pig effects and surrogate variables in the scale of log_2_(CPM) revealed samples were clustered by time and treatment.

Differential expression analysis was performed by comparing the level of gene expression at 2 h or 6 h post LPS and Poly (I:C) treatments compared to the non-stimulated AM at the corresponding time point. In total, we identified 5,760 genes that were differentially expressed (DE) between non-stimulated and stimulated AM (Supplementary File S2). The number of up-regulated DEGs were larger than that of down-regulated DEGs after stimulation. In response to LPS treatment 3,295 DEGs were identified, of which 926 genes were differentially expressed (DE) at 2 h and 2,369 genes were DE at 6h. In response to Poly (I:C) treatment 2,465 DEGs were identified, 818 of them were DE at 2h and 1,647 at 6h (Table 1). Although both treatments induced a similar transcriptional response at 2h and 6h, groups of DEGs was due to a combined treatment and time effect (Figure 1C). Next, we selected orthologous LPS-response genes in mouse bone marrow-derived macrophages (MBMM) at 6h, human monocyte-derived macrophages (HMDM) at 6h, porcine monocyte-derived macrophages (PMDM) at 7h (Schroder et al., 2012; Kapetanovic et al., 2013) to perform cross-species comparison with LPS-DEG in AM. The Spearman correlation test show significant correlation in LPS-response genes in all species, with PMDM (*R*^2^ = 0.79, *P* < 2.2x10^–16^) the most correlated LPS-gene response followed by HMDM (*R*^2^ = 0.49, *P* < 2.2x10^–16^) and MBMM (*R*^2^ = 0.43, *P* < 2.2x10^–16^) (Supplementary Table S3 and Supplementary Figure S3). Then, we determined using Wilcoxon rank-sum test that PMDM and HMDM LPS-response genes were not statistically (P 0.1) different to AM LPS-response genes, while MBMM genes were statistically different (P 0.001) (Supplementary Table S3).

**TABLE 1:** Summary of differential gene expression results of alveolar macrophages stimulated with LPS and Poly (I:C) at 2 and 6 h.

	LPS		Poly(I:C)	
	2h	6h	2h	6h
Down-regulated	328	1063	239	690
Up-regulated	598	1306	579	957
Total	926	2369	818	1647

**FIGURE 2:**
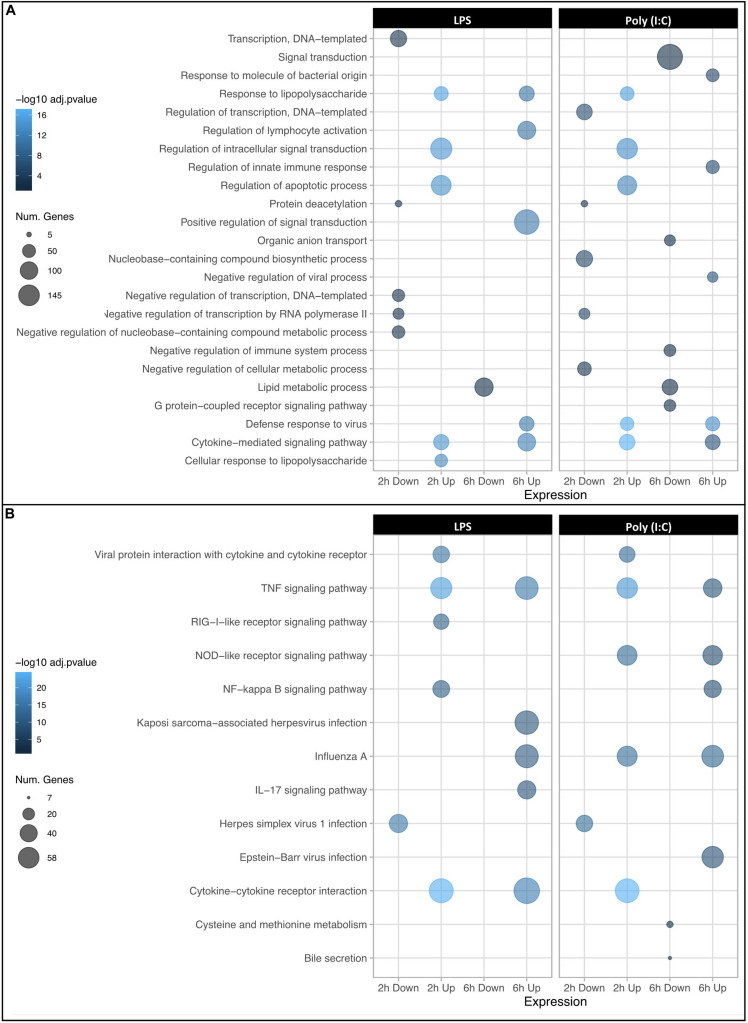
Dot plots showing GO term enrichment for DEGs. The top 5 most significantly enriched biological processes **(A)** and KEGG pathways **(B)** are displayed. The X axis corresponds to downregulated and upregulated genes at 2 and 6 h post stimulation, and Y axis represents the GO terms. The size and color of the dots corresponds to the number of DEGs associated with the GO terms and pathways and the *P*-values of hypergeometric tests, respectively.

As an initial validation of the RNAseq data, the estimate of Fold change ratio due to treatment of the five inflammatory genes at both 2 and 6 h was compared to the values reported above for qPCR. The RNAseq data was significantly correlated with that seen for the q-PCR data (*R*^2^ = 0.73, *P* < 0.0014) (Supplementary Figure S4). The DEG were then annotated for enrichment of Gene Ontology (GO) terms and KEGG pathways (Supplementary File S3). As expected, the Top 5 GO terms of upregulated genes at 2h and 6h due to either LPS or Poly (I:C) stimulation were mainly classified into terms associated with biological functions, such as immune system activation by the inflammatory response (e.g., cytokine-mediated signaling pathway, response to LPS, defense response to virus among others). Notably, at 6h a greater number of genes DE by LPS were enriched for the biological functions “positive regulation of signal transduction” and those DE by Poly(I:C) were enriched by “signal transduction” (Figure 2A). In addition to these enriched biological functions, genes with increased RNA abundance in response to both stimulations at 2h and 6h were highly enriched for TNF signaling pathway genes (Figure 2B and Supplementary File S3). Moreover, TLR signaling pathway and RIG-I like receptor signaling pathway were enriched at 2h and 6h in both treatments. Genes with decreased RNA levels in response to either treatment were enriched for several GO terms including “negative regulation of transcription” mainly at 2h and “lipid metabolic processes” at 6h. Interestingly, we found a group of downregulated genes (*ING2, JDP2, SAP30, SIRT4, SMARCAD1, ZBTB7B*) that were enriched for “protein deacetylation” function in both treatments at 2h. Histone deacetylases (HDACs) had an opposite expression patterns: genes such as *HDAC5* (down- LPS 6h) and *HDAC7* (down- LPS 6h) had decreased RNA levels, meanwhile genes such as *HDAC6* (up- Poly (I:C) 6h), *HDAC9* (up- LPS and Poly (I:C) 6h) and *SIRT1* (up- LPS and Poly (I:C) 2h and 6h) were found with increased levels of RNA. Contrary to the biological function enrichment observed, genes with decreased RNA levels were for just a few KEGG pathway terms such as “Herpes simplex virus 1 infection” (LPS, Poly (I:C) 2h) and “cysteine and methionine metabolism” (Poly (I:C) 6h). In summary, DEG and GO analysis indicated a similar biological response to the two stimulations, as genes with increased RNA levels upon either LPS or Poly (I:C) were enriched for GO terms related to the activation of the immune response. However, some specific responses to stimulation of TLR4 or TLR3 and RIG-I like receptor were detected that have not previously been annotated as specific for bacterial and viral infection in swine.

### Defining Both Unique and Common RNA Responses to LPS and Poly (I:C)

To identify the common RNA transcriptional responses via TLR4 and TLR3, RIG-I/MDA-5, we compared the DEG (treatments/controls) lists between treatments at 2h and 6h. All genes with similar response to the two treatments were also concordant between time of treatments except for *SLCO4A1* and *VDR*, which were higher in LPS and lower in Poly (I:C) treatment at 6h. The full description of the number of common, unique genes and their GO terms is available in the Supplementary Figure S5 and Supplementary File S4).

To better understand the differences in RNA expression induced by the LPS versus Poly (I:C) treatment, we performed a pairwise comparison between treatments with the aim of identifying genes specifically induced by LPS versus Poly (I:C), and vice versa (Supplementary File S5 and Supplementary Figure S6). Next, we compared the DEG (treatment/control) with the pairwise comparison between treatments and timepoints to determine which genes had statistically significant responses to only one of the two treatments or at only one timepoint. For treatments, we identified 29 and 102 genes that were only DEG by LPS treatment at 2h and 6h and were statistically different to Poly (I:C) treatment. At the same time, we identified 22 and 19 DEG genes that were only DEG following Poly (I:C) treatment at 2h and 6h and were statistically different than LPS treatment response (Table 2). For timepoint differences, we observed 53 and 358 genes that were only DEG at 2h and 6h by LPS treatment and were statistically different to the RNA level at the other timepoint. Finally, we noted 79 and 184 genes that were only DEG at 2h and 6h by Poly (I:C) treatment and were statistically different to 6h and 2h, respectively (Table 2). Taken together, this analysis strongly indicates that the majority of DEG in response to bacterial (LPS) or viral (Poly (I:C) mimics were consistent between the two stimuli. However, we could identify a set of genes that were DE for one treatment only at either 2h or 6h of stimulation.

**TABLE 2:** List of DEGs that highly respond exclusively to stimulation by LPS and Poly (I:C).

Treatments comparison	Genes
Unique DEG in LPS treatment at 2h + Highly expressed in LPS than Poly(I:C) treatment at 2h	*SKIL, PLK3, TMEM88, BCL3, PIM1, HILPDA, PLAGL2, BHLHE40, IL10RA, PTAFR, VEGFA, GDF15, SLC16A6, NFKBIE, G0S2, GPR65, PORCN, GPR132, EBI3, CDK5R1, NFKBID, SOX4, IER3, ZC3H12A, AMCF-II, NOS2, TNFAIP2, ENSSSCG00000008954, IL12B*
Unique DEG in Poly(I:C) treatment at 2h + Highly expressed in Poly(I:C) than LPS treatment at 2h	*ZFP90, RF00045, MEPCE, MTURN, STN1, ENSSSCG00000024973, RNASEL, CFAP74, KLHL25, TNFSF13B, FKBP14, B3GNT5, C5orf30, CH25H, UBA7, PALMD, FAM46A, CDHR4, CYP4A24, CEBPE, TNFSF10, AMOTL2*
Unique DEG in LPS treatment at 6h + Highly expressed in LPS than Poly(I:C) treatment at 6h	*MGAT5, SH2D1B, SNAPC1, ENSSSCG00000035650, NT5C, NFIL3, SLC16A6, PCK2, NT5DC2, GPR18, EHBP1L1, VEGFA, PGM2, AGPS, TPMT, DUOXA2, CHD9, PFKFB3, MSH2, CDC42EP2, SLC7A5, RAB7B, ENSSSCG00000035736, PTGS2, AHCTF1, IGSF3, CHST15, PLK3, ARSG, TMEM2, HBEGF, MCTP2, FOSL2, PXYLP1, SQLE, SEMA3C, CXCR4, ENSSSCG00000033183, GPR160, OSBPL3, GJA1, ENSSSCG00000036622, PAPSS2, CALD1, STAT5A, SAMSN1, PRKCE, IL1RAP, RAI14, FAM177A1, FOSL1, TNFSF15, SLC1A2, TRIM36, UPP1, PLAUR, MAMLD1, HILPDA, CCL22, IRAK3, ENSSSCG00000003079, CD72, PIM2, INHBA, F3, SOWAHA, ENSSSCG00000031023, HTR7, FFAR2, ARG2, SYN1, OSM, BATF3, FSTL1, ZNF648, TGM3, PLXNA1, TNFRSF6B, TNFAIP2, MFSD2A, SOCS3, TMEM120B, ENSSSCG00000036117, CHI3L2, ADORA2A, MMP2, INHBB, RASGRP1, CDCP1, ZC3H12A, ENSSSCG00000004572, SOX4, ENSSSCG00000008954, GFPT2, EBI3, IL12B, AMCF-II, CSF2, SERPINB2, CCL20, IL23A, CSF3*
Unique DEG in Poly(I:C) treatment at 6h + Highly expressed in Poly(I:C) than LPS treatment at 6h	*LOXL3, DAPP1, SEMA4A, GFOD1, IL27RA, SLC24A4, PALMD, MPZL1, MTURN, S100A2, HMGN5, STAC2, CYP4A24, WNT9A, AMOT, P2RY13, CCL2, ENSSSCG00000023014, TEK*

*Highly expressed genes in each treatment were defined by pairwise comparison between treatments.*

### Specific HMs Are Associated With Gene Expression in Porcine AM, and Are Consistent With Those Reported for Selected Human Macrophage Expressed Genes

**FIGURE 3:**
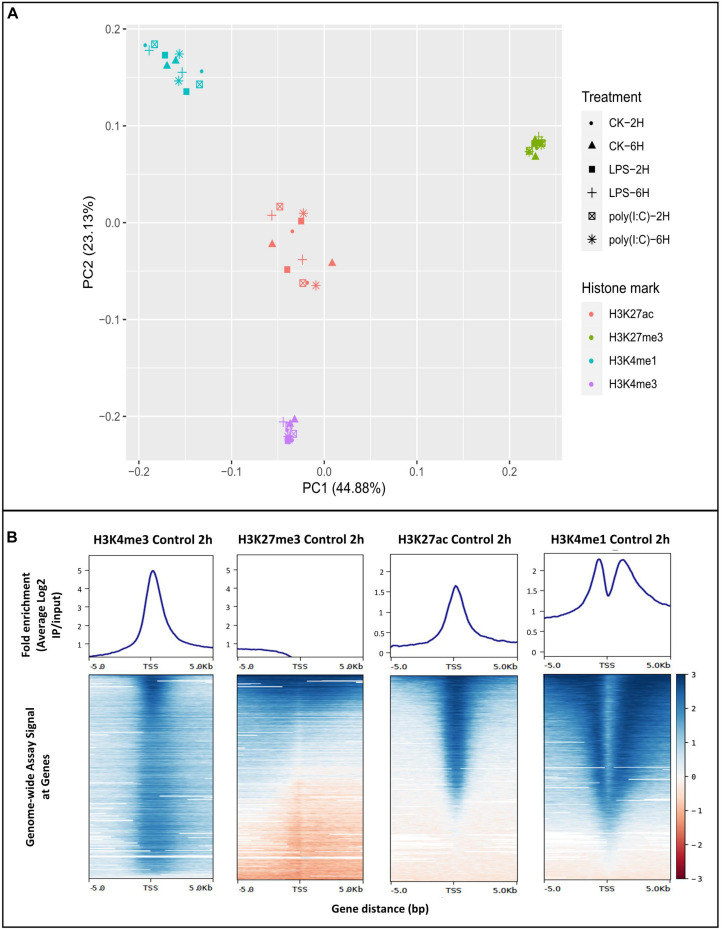
Chromatin histone modification analysis of porcine alveolar macrophages. **(A)** Principal component analysis plot of histone ChIP-seq samples. **(B)** The average genome wide histone fold enrichment (average log_2_ IP/input) near TSS (± 5.0 Kb) was calculated for each individual histone mark. Non-stimulated alveolar macrophages at 2 h were displayed in the figure as representative.

To explore regulatory mechanisms associated with modified histones on AM gene expression in response to LPS or Poly (I:C) at 2h and 6h, we performed ChIP-seq. The four histone marks analyzed in this study included all recommended FAANG HMs (Tuggle et al., 2016; Giuffra et al., 2019): H3K4me3 (promoter regions), H3K27me3 (associated with Polycomb repression), H3K27ac (active enhancer and promoter regions) and H3K4me1 (promoter and enhancer regions). The ChIP-seq produced an average of 35,351,781 of uniquely mapped reads per sample (range: 10,649,687-74,965,735 reads) for narrow mark H3K27ac. We produced an average of 77,226,790 of uniquely mapped reads per sample (range: 20,686,265-117,143,376 reads) for broad marks H3K4me1, H3K27me3 and H3K4me3. For all marks, the ENCODE recommended guidelines were surpassed for ChIP-seq quality (Landt et al., 2012). Furthermore, over 94% of the reads aligned to the *Sus scrofa* reference genome (range 66–99%). The marks H3K27me3 and H3K4me3 had the highest number of sequencing reads across all samples analyzed (average: H3K27me3: 105,368,665 of uniquely mapped reads, H3K4me3: 95,087,325 of uniquely mapped reads) as well as the highest alignment rate to the genome (average: H3K27me3-99%, H3K4me3-97%, H3K4me1-95%), followed by narrow mark and H3K27ac (85%) (Supplementary File S6). As expected, AM samples clustered primarily by histone modification into repressive H3K27me3 and activating marks H3K4me3, H3K4me1 and H3K27ac based on principal component analysis (Figure 3A) or correlation (Supplementary Figure S7) and this separation was independent of the genetic differences across biological replicates (less than 37%) (Supplementary Table S4).

On average, we identified a total of 74,812, 51,443, 40,046 and 27,145 (enriched regions compared to input DNA) within replicates of H3K4me1, H3K4me3, H3K27ac, and H3K27me3, respectively (Supplementary Figure S8). Enriched regions for H3K27ac and H3K27me3 reported here are comparable with Qiao et al., 2016 in resting macrophages derived from monocytes (35,272-H3K27ac and 19,784-H3K27me3 peaks) (Qiao et al., 2016). Highly reproducible peaks using IDR method were define and are available in the Animal Genome repository^10^. ChIPQC package was used to assess the quality of the ChIP-seq (Carroll et al., 2014), and the relative cross-coverage score for all samples indicated good enrichment (Supplementary File S7). Another quality control measure, the fraction of reads in peak regions (FRiP) (Landt et al., 2012) was calculated to estimate S/N or enrichment. The average FRiP values obtained were over 46% (Supplementary File S7), which is much higher than minimum used for ENCODE (1% FRiP, Landt et al., 2012). Overall, ChIPQC analysis demonstrated high quality and enrichment for all histone marks analyzed.

**FIGURE 4:**
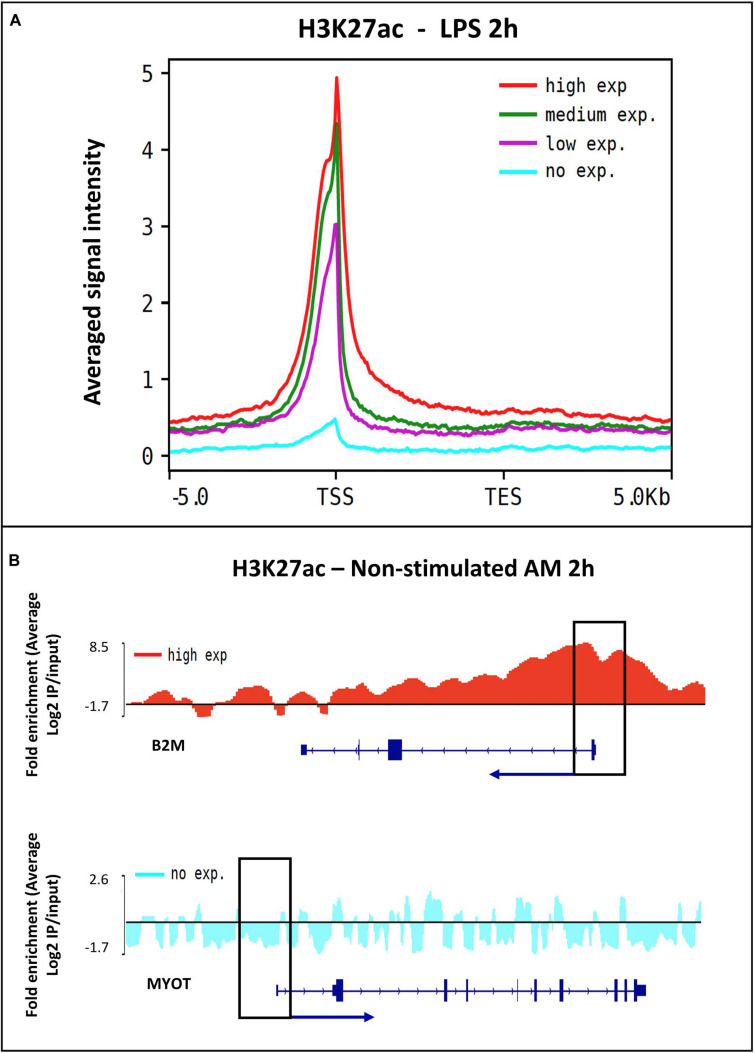
The relationship between H3K27ac signal and gene expression. **(A)** Histogram of the H3K27ac signal intensity and all expressed genes in alveolar macrophages stimulated with LPS treatment at 2 h. Genes were divided into four quartiles, high expression (red), middle high (green), middle low (purple) and low expression (blue). **(B)** Peak visualization using the Integrative Genomics Viewer (IGV) of a highly expressed gene ACTB and a low expressed gene MYOT in non-stimulated alveolar macrophages.

To confirm the distribution pattern of the ChIP-seq peaks, we analyzed the histone mark reads distribution across transcriptional start site (TSS) (−/+5.0 Kb) genome wide for all samples. High enrichment of active histone marks H3K4me3, H3K4me1 and H3K27ac was observed at TSS regions (Figure 3B). In addition, the nucleosome free region at TSS was observed with a signal drop in H3K4me1 enrichment. As expected, the repressive mark H3K27me3 did not have enrichment relative to input near TSS (Figure 3B). Classification of the histone mark distribution among genomic features showed that the histone marks H3K27me3, H3K27ac, H3K4me3 and H3K4me1 were mainly enriched in promoter regions (66.7, 56.6, 54.9, and 35.8%, respectively), followed by distal intergenic and intronic regions (Supplementary Figure S9 and Supplementary File S7). Next, we validated the association of the H3K27ac signal at promoter regions through sub-setting all genes into quartiles based on expression level. Promoter enrichment of H3K27ac was highly associated with the level of gene expression (Figure 4A). Figure 4B illustrates high enrichment of H3K27ac at a gene highly expressed in AM, beta-Actin (*ACTB*), and no enrichment at the Myotilin gene (*MYOT*) which was not expressed in AM. These results demonstrate that ChIP-seq data consistently detected biologically relevant histone marks across different sample treatments as well as replicates (Supplementary Figure S10).

To further explore and validate these histone modification data, we compared the distribution of the active histone modifications H3K4me3, H3K27ac, and H3K4me1 between non-stimulated human bone-marrow-derived macrophages and porcine AM. We observed a similar histone modification distribution between human and pig for expressed genes in macrophages involved in TLR signaling pathway such as *CD40*, *CD14*, *RELA*, *CCL3L1* and *TNF* as well as the control (non-expressed) F9 gene (Supplementary Figure S11).

### Chromatin State Map of Porcine Alveolar Macrophages Predicts Regulatory Regions Genome-Wide and Was Resilient Up to Six Hours After Inflammatory Stimulation

**FIGURE 5:**
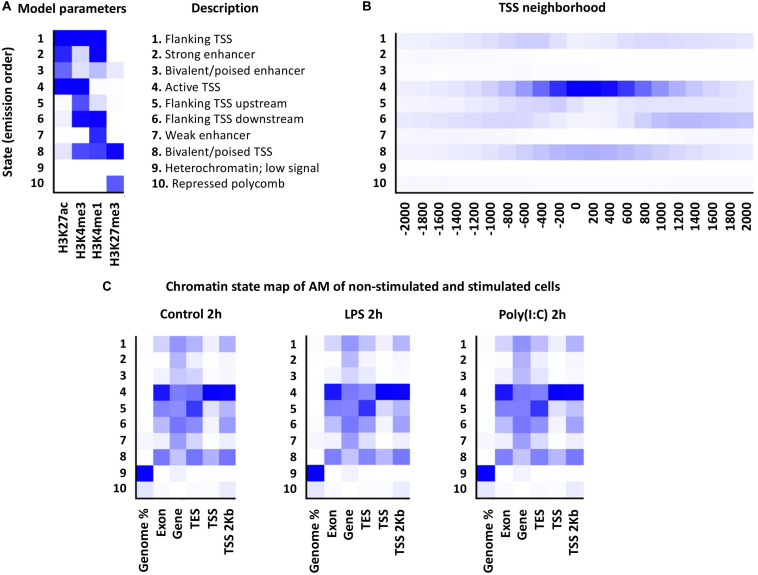
Chromatin states of porcine alveolar macrophages. **(A)** At left is shown a heatmap of the emission parameters, each row corresponds to a different state, and column for each histone mark. The darker blue color corresponds to a greater probability of observing the histone mark. At right is shown the description of the specific chromatin state **(B)** The TSS neighborhood heatmap shows the overlap enrichment for each state for each 200-bp bin within 2 kb around a set of TSSs. **(C)** Heatmap showing the emission parameters of non-stimulated and stimulated alveolar macrophages with LPS or Poly (I:C) at 2 h. The heatmap displays the overlap enrichment of the histone mark on the current pig genome annotation.

Using the ChromHMM software, the first porcine AM chromatin state map of non-stimulated and LPS or Poly (I:C) stimulated cells was created. Implementation of the Hidden Markov Model (HMM) uses histone mark data to represent different hidden states of the “chromatin states” based on the presence/absence of multiple histone marks (emission parameters) and the special constraints of how these histone mark combinations occur relative to each other across the genome (Ernst and Kellis, 2017). According to the four histone mark combinations used for AM, 10 chromatin states representing the combinatorial distribution of HMs were established: transcription start sites (TSS) regions (states 1, 4, 5, 6 and 8), potential enhancers (states 2, 3 and 7), repressed polycomb (state 10) and low signal (state 9) (Figure 5A). Chromatin states of all treatments and timepoints are available in the Animal Genome repository^10^. Distribution of predicted chromatin states in the current porcine genome annotation (*Sus scrofa* 11.1, Ensembl, version 90) was as expected. Regions around 2 Kb of TSS were enriched specifically for chromatin states 1, 4, 5, 6 and 8 corresponding to promoter regions and CpG islands (promoter regions) (Figure 5B). Notably, the histone marks distribution around TSS of DEG did not show large-scale changes in histone mark regions due to LPS or Poly (I:C) treatment at 2h and 6h (Figure 5C).

### Substantial Changes of H3K27ac Modification Levels Immediately After TLR4 and TLR3/RIG-I/MDA-5 Pathway Stimulation

The chromatin state analysis showed that AM chromatin did not undergo broad changes in the chromatin states at 2h or 6h. However, differences in the level of histone enrichment (differential histone modification regions, DHMRs) are associated strongly with gene expression changes (Shen et al., 2013). Therefore, the changes of the HMs in the genomic regions between non-stimulated and stimulated AM was calculated (| log_2_ FC| > 1, FDR < 0.05). The H3K27ac was the histone mark with more DHMRs after LPS or Poly (I:C) stimulation, followed by H3K4me3. In contrast, H3K4me1 and H3K27me3 enrichment levels were not modified by stimuli. In total, 10,290 DHMRs in all comparisons; 6,944 DHMRs between non-stimulated and stimulated AM, 1,097 between treatments type and 2,249 between timepoints (Table 3 and Supplementary File S8). Enrichment level of both H3K27ac and H3K4me3 increased 2h after LPS or Poly (I:C), and then decreased at 6h. In summary, a high number of DHMRs in AM after LPS or Poly (I:C) stimulation were detected, with the H3K27ac as the most dynamic histone mark, as reported by others (Novakovic et al., 2016; Borghini et al., 2018; Daskalaki et al., 2018), followed by H3K4me3.

**TABLE 3:** DHMRs of alveolar macrophages stimulated with LPS and Poly (I:C) and integration with DEG (underlined values represent significant enrichment of DHMRs at DEG promoters, hypergeometric test, *p-*value < 0.05).

Histone mark	Comparision	DHMRs				Integration DHMRs at promoter regions of DEG			
		DHMRs		DHMRs at promoter regions		Down		Up	
		Down	Up	Down	Up	DEG	DHMRs + DEG	DEG	DHMRs + DEG
H3K27ac	LPS 2h – C2h	106	1196	20	356	328	1	598	91
	Poly(I:C) 2h – C2h	547	2986	152	998	239	9	579	232
	LPS 6h – C 6h	250	454	50	136	1063	16	1306	78
	Poly(I:C) 6h – C 6h	245	888	50	297	690	8	957	158
	Poly(I:C) 2h – LPS 2h	127	826	27	234	61	3	42	7
	Poly(I:C) 6h – LPS 6h	49	70	11	18	179	3	62	2
	LPS 6h – LPS 2h	309	31	98	19	542	4	586	1
	Poly(I:C) 6h – Poly(I:C) 2h	1505	325	473	85	373	30	287	3
H3K4me3	LPS 2h – C2h	0	52	0	20	328	0	598	6
	Poly(I:C) 2h – C2h	0	47	0	26	239	0	579	10
	LPS 6h – C 6h	4	79	4	43	1063	2	1306	25
	Poly(I:C) 6h – C 6h	16	74	2	32	690	0	957	17
	Poly(I:C) 2h – LPS 2h	1	3	1	1	61	0	42	0
	Poly(I:C) 6h – LPS 6h	20	1	6	0	179	4	62	0
	LPS 6h – LPS 2h	0	51	0	19	542	0	586	7
	Poly(I:C) 6h – Poly(I:C) 2h	3	25	2	11	373	0	287	2

### Changes of H3K27ac Enrichment on Promoter Regions Is Associated With Gene Expression Changes Induced by LPS and Poly(I:C)

To explore in more detail the link between epigenetic regulation and gene expression, the ChIP-seq and RNA-seq results were integrated to determine if DHMRs have a role in the AM transcriptional response to LPS or Poly (I:C). We assigned the DHMRs to the nearest known transcripts (5kb flanking TSS) in the current porcine genome annotation, and then compared these regions with DEG abundance (Hypergeometric distribution test, *P* < 0.05). After integration, the increase of H3K27ac and H3K4me3 at the promoter region of upregulated genes (range: 78-232 DHMRs-H3K27ac; 6-25 DHMRs-H3K4me3) was higher than the decrease of H3K27ac and H3K4me3 at the promoter region of downregulated genes (range: 1-16 DHMRs-H3K27ac; 2-4 DHMRs-H3K4me3). Furthermore, the integration showed a few H3K27ac and H3K4me changes over time and across treatments (Table 3 and Supplementary File S9).

Although an association between H3K4me3 and some DEG was found, only the list of genes with an increase of H3K27ac at promoter regions and higher levels of RNA included sufficient genes to be considered for further analysis. H3K27ac was significantly positively correlated with gene expression changes, although the correlation observed was weak at 2h and moderate at 6h for both treatments (LPS 2h, *R*^2^ = 0.23, *P* = 0.02; LPS 6h, *R*^2^ = 0.77, *P* < 2.2x10^–16^; Poly(I:C) 2h, *R*^2^ = 0.45, *P* < 7x10^–16^; Poly(I:C) 6h, *R*^2^ = 0.68, *P* < 2.2x10^–16^) (Supplementary Figure S12). GO term analysis (Supplementary File S9) revealed that upregulated genes which showed increase of H3K27ac at promoter regions had enriched biological functions for immune system activation by the inflammatory response (e.g., cellular response to lipopolysaccharide, pattern recognition receptor signaling pathway, defense response to virus). A KEGG pathway analysis of these increased H3K27ac-associated DEG was also performed. Both LPS or Poly (I:C) treatment for 2h and 6h shared KEGG pathways related to signaling pathways relevant to immune response. These pathways included Influenza A, Cytokine-cytokine receptor interaction, TNF signaling pathway, Toll-like receptor signaling pathway, NF-kappa B signaling pathway, RIG-I-like receptor signaling pathway, NOD-like receptor signaling pathway, JAK-STAT signaling pathway, IL-17 signaling pathway, Th17 differentiation, among others (Supplementary Table S5). Although enriched KEGG pathways were similar between treatments, we found three different groups of genes enriched for KEGG pathway membership between the treatments. The first group included some genes that showed DHMRs only for one treatment, yet were DE in response to both treatments (*PPP3CC, STAT5A, NFκB2, IFNLR1, CXCL2, IL1A, IFN-ALPHAOMEGA, PANX1, TXNIP, FASLG, TRAF3, PIK3CD, IFIH1, JAK2, MAPK14, NOD2, MLKL, CD40, IL27RA, IL10RB, TIRAP* and *CD80*). The second group was shaped by DEG that responded to only one treatment, yet showed DHMRs for the same treatment (*SLA-DQB1* and *TNFSF13B*, Poly (I:C) 2h; *FOSL1*, LPS 6h; *AMCF-II* LPS 6h), Finally, the third group included genes that were DE and showed DHMRs for both treatments (*CXCL8, CCL5, CSF3, TNF, NFκB1, LTA, IL4R, CFLAR, TRAF1, SOCS1, CXCL10, DDX58, TNFAIP3, EDN1, IRF1, BCL2A1, ADAR, MX1, TRIM25, RSAD2, CCL3L1, IL27, MAP3K8, DHX58*).

**FIGURE 6:**
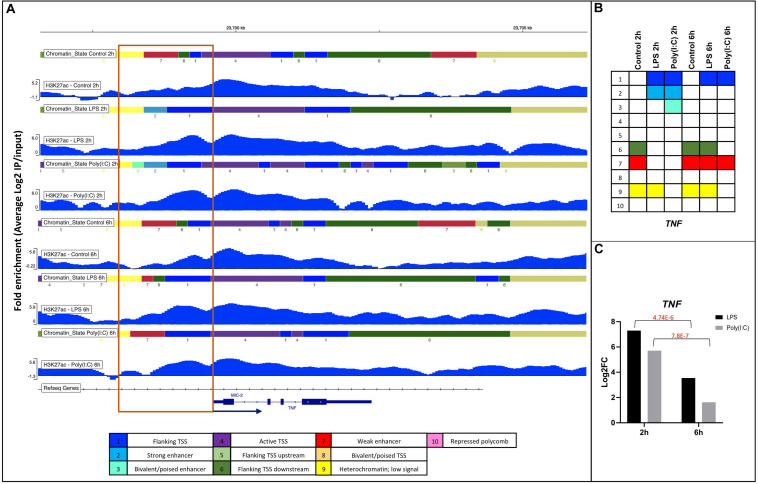
Changes in chromatin state of the *TNF* gene in response to LPS and Poly(I:C) at 2 h and 6 h. **(A)** IGV screenshots showing DHMRs-H3K27ac with chromatin states around 1kb of promoter regions of *TNF* gene in respond to treatments. Annotation of the chromatin states is shown as legend on the bottom and as **(B)** summary table. **(C)** Gene expression values of *TNF* gene from RNA-seq of stimulated AM.

As DHMRs-H3K27ac were observed near the promoter regions (5kb flanking TSS) of DEG involved in immune system activation, we anticipated observing predicted chromatin state changes of promoter regions associated with immune activation after stimulation. For this Integrative Genomics Viewer (IGV) analysis, we chose the increase of DHMRs-H3K27ac 1kb up and downstream of the TSS for upregulated genes involved in immune KEGG pathways mentioned above (56 genes) (Supplementary File S10). A total of 41 DEG were found with DHMRs-H3K27ac at their promoter regions; all were visualized on IGV. Only 20 of 41 genes showed changes in chromatin states related to the level of gene expression, e.g., weak enhancer to strong enhancer (low gene expression in controls to high gene expression in treatment). The chromatin state changes that we observed were diverse and the borders and type of predicted states varied (Supplementary File S10). In Figure 6 and Supplementary Figure S13, we display two clear examples of such chromatin state change, where the conversion of weak enhancer to strong enhancer function near the promoter was corroborated by the increase of the gene expression of *TNF* and *CCL3L1* after stimulation.

### Transcription Factor Binding Motif (TFBM) Analysis Reveal the Association of H3K27ac Enrichment and TF Binding Sites

**FIGURE 7:**
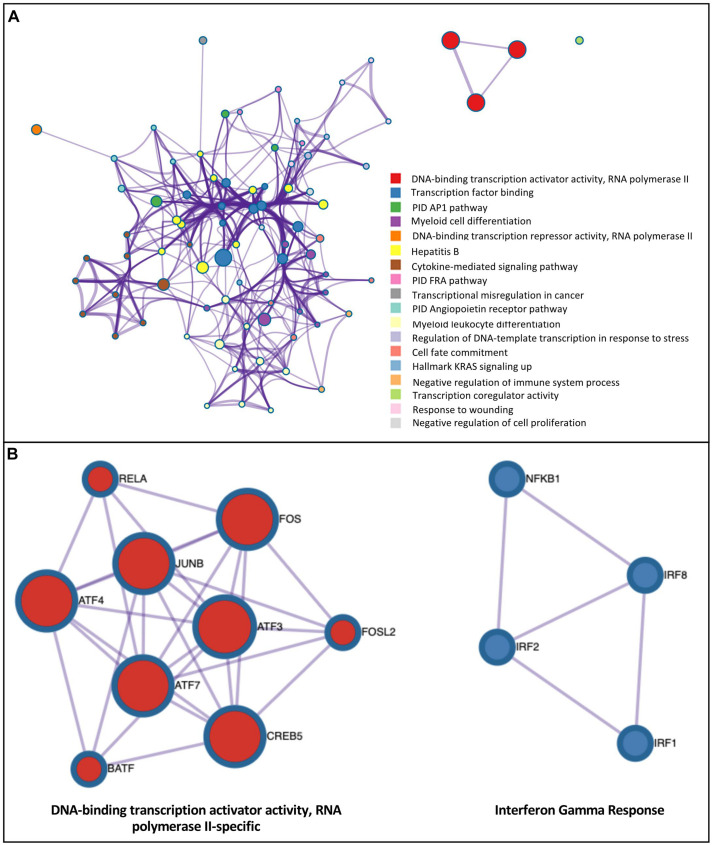
Integrated analysis of H3K27ac modification and RNA expression response to inflammatory stimuli demonstrates enrichment of binding motifs for TF induced by these stimuli. **(A)** Ontology enrichment clusters of upregulated transcription factors that were induced by the LPS-2 h treatment whose TFBM was enriched in regions with a gain of H3K27ac modification of DEG. The most statistically significant term within similar term clusters (enclosed by dotted lines) was chosen to represent the cluster. Term color is given by cluster ID and the size of the terms is given by –log10 *P*-value. The stronger the similarity among terms, the thicker the edges between them. **(B)** PPI network of upregulated transcription factors with enriched H3K27ac motifs of DEG for LPS-2h treatment. A unique color is assigned to each MCODE network.

To investigate potential regulatory pathways illuminated by H3K27ac marks in AM, we tested whether the H3K27ac peaks contained an enrichment of specific transcription factor binding motifs (TFBM) using HOMER (Heinz et al., 2010). We tested different defined groups of H3K27ac-associated regions, including: (a) H3K27ac enrichment in all treatments and timepoints; (b) H3K27ac enrichment within a defined chromatin state “active promoter” region around the TSS of DEG; and (c) genome-wide H3K27ac differential peak regions in response to treatments. Only the TFs expressed in AM were selected for these analyses (Supplementary File S11). Group (a): Using the H3K27ac enrichment (peaks) genome-wide data across treatments and time points, we found on average 62 significantly enriched TFBM (Supplementary Table S6). As expected, based on the similarity of H3K27ac peak regions across conditions, the majority of binding motif and associated TFs predicted were common in all treatments and timepoints. Motifs for several relevant TFs, such as PU.1, AP-1 family members (*Jun*, *JUNB/D*, *FOS*, *FOSL2*, *BATF*, *ATF2/3/4/7* and *MAFB/F/K*) interferon regulatory factors (*IRF1/2/3/8*) and *NF*κ*B-65* (*RELA*), were identified. Interestingly, the motif for *NF*κ*B p50/p52* (NFκB1) was enriched only in LPS and Poly(I:C) at 2h using the H3K27ac peaks (Supplementary File S11). Group (b): Using the H3K27ac-enriched regions of defined “active promoters” taken from the chromatin state analysis above, we obtained on average 20 and 10 significant TFBM in upregulated and downregulated genes in response to treatments (Supplementary Table S6). As seen in the H3K27ac enrichment genome-wide analysis, promoter regions showed consistency of binding sites and associated TFs predicted in all treatments and timepoints. TFBMs enriched in promoter-H3K27ac regions were highly enriched for DNA binding transcription activator/repressor activity, and terms related with the activation of the immune system such as regulation of cytokine production (Supplementary File S12). Group (c): To further investigate functional properties of differential H3K27ac peaks, we tested whether the differential peaks were enriched for specific TFBM across treatments. We detected on average 44 and 14 TFBM in up and down regulated H3K27ac peaks, respectively (Supplementary Table S6). TFBMs enriched in DHMR-H3K27ac regions were associated with several terms related to bacterial and viral response, in addition to general TF functional terms such as transcription factor binding. For example, in LPS-2h upregulated DEG, these terms included cytokine-mediated signaling pathway, AP1 pathway, negative regulation of immune system process (Figure 7A), TNF signaling via NFκB, response to interferon-gamma among others (Supplementary File S12). A further analysis of these TFs whose motifs are enriched in DHMR-H3K27ac regions using protein-protein interaction (PPI) data in the Molecular Complex Detection (MCODE) algorithm, identified densely connected functions such as DNA-binding transcription factor activity and interferon gamma response (Figure 7B). The majority of interconnected TFs in this PPI analysis were consistent across treatments.

Finally, we used the RNA-seq data to investigate whether TF whose motifs are enriched in DHMR-H3K27ac regions are themselves regulated by the LPS and Poly(I:C) treatments. We identified 8, 6, 7 and 5 upregulated RNAs for transcription factors with enriched motifs in H3K27ac peak regions increased by LPS-2h, LPS-6h, Poly(I:C)-2h and Poly(I:C)-6h treatments, respectively (Table 4). These upregulated TFs included *IRF1* and *NKFB1*, both increased for each treatment and both timepoints. *NFE2L2* (also called *NRF2)* increased at 2h for LPS and at 6h for both treatments. *CREB5*, *IRF4* and *STAT1* were increased in response to LPS, while *EHF*, *KLF5* and *ELF1* responded only to Poly(I:C). *IRF8*, *RELA* and *PRDM1* responded at 2h, and *IRF2* only at 6h, for both treatments. No downregulated TF with motif enrichment in decreased H3K27ac peak regions was found. Using this combinatorial approach of HMs, TFBM and gene expression analyses, we were able to find TFs that were induced by the treatments whose TFBM was enriched in regions with a gain of H3K27ac modification. For example, pro-inflammatory (*STAT1*, *IRF1*/*4*/8, *RELA*, *NFκB1*) and anti-inflammatory TFs (*NFE2L2*, called *NRF2*) were identified, as well as an antiviral TF (*ELF1*). Seifert et al., 2019 reported that expression of the TF *ELF1* inhibits the infection of eight diverse RNA and DNA viruses independent from the action of the type I interferons (Seifert et al., 2019). Collectively, these findings provide integrated evidence identifying TFs linking an increase of gene expression with increased presence of H3K27ac in inflammatory processes within AM. Thus, transcriptional and epigenetic regulation are interconnected in the response of porcine AM against viral or bacterial stimuli.

**TABLE 4:** Upregulated transcription factors with predicted DNA binding motifs in regions enriched for H3K27ac in stimulated AM.

LPS		Poly(I:C)	
2h-Up	6h-Up	2h-Up	6h-Up
CREB5			
IRF4	IRF4		
	STAT1		
		EHF	
		KLF5	
			ELF1
IRF1	IRF1	IRF1	IRF1
NFKB1	NFKB1	NFKB1	NFKB1
IRF8		IRF8	
RELA		RELA	
PRDM1		PRDM1	
	IRF2		IRF2
NFE2L2	NFE2L2		NFE2L2

## Discussion

Alveolar macrophages have broad effector functions to maintain lung health, and have the ability to respond to a variety of PAMPs, responding relative to the stimulus (Hoeksema and de Winther, 2016). Immune cells, including AM, have transmembrane TLRs (TLR4) and intracellular receptors (TLR3 and - RIG-I/MDA-5) to sense microbial molecules and activate the immune system (Brubaker et al., 2015). Stimulation of TLRs promotes chromatin remodeling by chromatin modifications (Foster et al., 2007; Lee et al., 2012). However, epigenetic regulation of the transcriptional response to TLR stimulation remains poorly understood in swine. In this study, we investigated the epigenetic state via histone modification on the transcriptional response of porcine AM to in response to LPS and Poly (I:C).

First, we evaluated the gene transcription changes at 2h and 6h following LPS or Poly (I:C) exposure. Our results show similarities to transcriptional responses in human, mice and swine in different types of macrophages (Huang et al., 2006; Schroder et al., 2012; Kapetanovic et al., 2013; Das et al., 2015; Pinilla-Vera et al., 2016; Igata et al., 2019). The increased expression levels of pro-inflammatory genes via *NFκB* were also observed in our porcine AM data set, demonstrating consistency with these previous studies (Huang et al., 2006; Kapetanovic et al., 2013; Das et al., 2015; Pinilla-Vera et al., 2016; Igata et al., 2019). It was of interest that the LPS-response in swine AM was substantially more similar to human responses than seen for mouse; however, it should be noted that the available such data for different species was derived from different types of monocytes. A more refined comparison would require common cell sources and culture conditions.

Part of the increased RNA expression after either stimuli may be explained by the downregulation of genes involved in negative regulation of transcription, such as *ZNF174*, a zinc finger protein (Williams et al., 1995). In our data we found the downregulation of several members of the zinc finger gene family, which bind to DNA and RNA to regulate transcriptional activity, increase expression of genes and stimulate a subsequent immune response (Lupo et al., 2013; Yang et al., 2015). We observed decreased expression of negative regulators of transcription, including decreased expression of genes involved in protein deacetylation, which has subsequent effects on chromatin remodeling and gene expression (Kapellos and Iqbal, 2016). For example, a gene downregulated in both treatments, *JDP2*, is classified within the protein deacetylation biological function. JDP2 protein acts as repressor of activation of transcription via *AP-1* (Tsai et al., 2016) which is involved in the inflammatory processes downstream of *NFκB* (Krappmann et al., 2004). Taken together, downregulation of negative regulators of transcription and genes classified in protein deacetylation pathways support the observed increase in gene expression and inflammatory response by porcine AM after LPS or Poly (I:C) exposure.

The majority of genes expressed at 2h and 6h in AM after LPS or Poly (I:C) exposure were common between stimuli (Supplementary Figure S5 and Supplementary File S4), which could be considered as a core response to bacteria and virus. Although both treatments induced a similar transcriptional response, it is not surprising given that TLR4, TLR3 and RIG-I/MDA-5 signaling pathways converge at *NFκB* activation (Kawai and Akira, 2007; Amit et al., 2009). This can explain the similar GO classification with LPS and Poly (I:C) stimuli. In spite of all common genes were concordant between treatments, *SLCO4A1* and *VRD* had different RNA levels in response to LPS (upregulated) and Poly (I:C) (downregulated) treatment at 6h (Supplementary Figure S5). Both *SLCO4A1* and *VRD* have been reported to be part of the RNA response to bacterial (Dower et al., 2008; Fiske et al., 2019) or viral infections (Petrovic and Piquette-Miller, 2010; Gotlieb et al., 2018). In addition, we observed differential expression in three members of the interferon (IFN) gene family *IFNA1*, *IFNB1* and porcine *IFN-ALPHAOMEGA* (Li et al., 2019). All three IFN genes were DE in both LPS and Poly (I:C) treatments and showed greater increase in RNA level after Poly (I:C) stimulation than after LPS stimulation. IFN genes are typically associated with viral infections; however, they may increase during bacterial infections (Sheikh et al., 2014; Boxx and Cheng, 2016). The RNA levels of *IFN* and others pro-inflammatory genes such as *IL6* and *IL12B* are negatively regulated by the activating transcription factor 3 (*ATF3*) (Labzin et al., 2015). *ATF3, IL6* and *IL12B* were overexpressed in our study for both treatments, and *ATF3* could be acting as a negative feedback-loop in porcine AM in response to bacterial and viral mimics (Gilchrist et al., 2006).

Besides the common RNA transcriptional responses observed for both treatments, unique transcriptional changes between LPS or Poly (I:C) were also found. The expression of swine leukocyte antigen (SLA) class II genes increased only in response to Poly (I:C). SLA molecules are involved in the adaptive immune response by presenting antigens to cognate T cells for effector functions, which is one of the functions of macrophages (Gordon and Plüddemann, 2017). Although pro-inflammatory transcriptional activators such as cytokines, chemokines and interleukins are typically induced by bacterial or viral infection (Mogensen and Paludan, 2001; Sanwald et al., 2015), we identified a group of transcription factors, inhibitors, chemokines and interleukins, such as *STAT3*, *NFκBID*, *NFκBIE AMCF-II* and *IL12B*, whose RNA levels increased in AM only in response only to LPS, which agrees with *STAT3* and *IL12B* transcription observed in human and murine macrophages (Meng et al., 2014; Ma et al., 2015; Liu X. et al., 2018). The function of these genes has been well documented as antimicrobial activity (*NOS2*) (Young et al., 2018) and inhibition of inflammation (*NFκBID* and *NFκBIE*) (Whiteside et al., 1997; Schuster et al., 2013). Interestingly, there is little information available about the neutrophil chemoattractant protein AMCF-II (alveolar macrophage-derived chemotactic factor-II) (Goodman et al., 1992). Poly (I:C) induced the B-cell activating factor of the TNF family *TNFSF13B* (also called *BAFF*) (Henley et al., 2008) and *DAPP1* (also called *Bam32*) which plays an important role in B cell receptor signaling, B cell survival and antigen presentation (Onyilagha et al., 2015), as well as *KLHL25* and *UBA7*, which are involved in the ubiquitin system for antigen processing and presentation (Vertegaal, 2011; Zhang et al., 2016).

A comparison of responses at each timepoint for both treatments showed an increase of transcriptional activity based on the number of genes with higher RNA levels at 6h than 2h. Previous studies have found an increase of transcription according to a temporal regulation of the pro-inflammatory response mediated by the induction of the *NFκB* and other transcription factor in macrophages (Gilchrist et al., 2006; Ravasi et al., 2007; Ramsey et al., 2008). At the same time, regulation of transcription is associated with changes in chromatin structure that include HMs. Furthermore, expression of histone acetylases and deacetylases and other genes involved in chromatin remodeling play a critical role in these processes (Kapellos and Iqbal, 2016). Thus, it is tempting to speculate that chromatin remodeling genes and pro-inflammatory transcription factors found in our study are important for the transcriptional response in AM.

Genome-wide histone modification profiling at 2h and 6h after LPS or Poly (I:C) stimulation showed few large changes in predicted chromatin states due to treatments in spite of the differential expression of genes involved in protein deacetylation and potential modification of the chromatin state and subsequent transcriptional response. The chromatin state map demonstrates a consistency of the chromatin structure in response to LPS and Poly (I:C) at 2h and 6h post stimulation. However, we could observe in non-stimulated AM (and seen in human macrophages via UCSC browser visualization) that expression of primary response genes was allowed by a permissive chromatin structure according to histone mark distribution (Supplementary Figure S11), as such genes were predicted to have strong/poised enhancers nearby active/poised promoters^10^ One interpretation is that the promoter regions and potential enhancers of pro-inflammatory genes are already active/poised before AM stimulation and therefore, the chromatin state analysis revealed few major changes on the chromatin state after LPS or Poly (I:C) stimulation. As AM are highly differentiated cells poised for effector function, perhaps this is not surprising, contrary to what is observed during hematopoiesis (Winter and Amit, 2014).

Active HMs (H3K27ac and H3K4me3) at promoter regions are typically associated with highly expressed genes (Lara-Astiaso et al., 2014). The H3K27ac is one of the most dynamic histone marks (Saeed et al., 2014) and as we anticipated, it was the histone mark with more changes after both stimuli at 2h and 6h (Table 3). H3K27ac is known to shape active promoters and enhancers by opening chromatin, thereby allowing the transcriptional machinery to assemble at the core promoter (Pradeepa, 2017). Several interesting observations were made when we specifically focused on the H3K27ac marker at inflammatory mediator and response genes with altered expression levels at 2h or 6h after LPS or Poly (I:C) stimulation. Given the effect of both treatments on the macrophage transcriptome, we could expect changes in the intensity of H3K27ac peaks genome-wide including promoter regions upon either stimuli (Supplementary File S8). Surprisingly, few of the H3K27ac peaks identified were unique to stimulated cells, as the majority of the identified peaks overlapped in genomic location between treatments. This indicates that these gene promoters are transcriptionally enabled prior to stimulation (Schmidt et al., 2016). However, we observed an increase of the enrichment following stimulation among such peaks at some genes that would indicate a further unpacking of chromatin (Table 3 and Supplementary File S8).

Our TFBM analysis using epigenetically marked regions demonstrated the value of determining genomic elements enriched for these functional marks. These analyses supported the finding of permissive chromatin in non-stimulated AM. The genome regions with high H3K27ac modification even in resting cells were enriched in TF known to mediate the macrophage inflammatory response to stimuli (Pham et al., 2012; Ha et al., 2019). These TF included PU.1, AP-1 members, interferon factors and RELA, whose motifs are in regions marked by H3K27ac prior to stimulation with LPS and Poly(I:C). PU.1 is consider to be a master macrophage transcription factor that initiates increased chromatin accessibility, allowing the binding of additional TFs such as *IRF*, *NFκB* and AP-1 factors necessary for response to these stimuli (Chen et al., 2020). Furthermore, we were able to detect evidence of new *NFκB1* binding motif enrichment in promoter regions with an increase in H3K27ac marks after both stimuli.

Although we observed DHMR of H3K4me3 and H3K27ac at 2h and 6h, it is important to note that genes with DHMR of H3K4me3 at promoter regions did not show the large changes in RNA level observed for H3K27ac DHMR. Thus, changes in H3K4me3 modification on promoter regions were not necessary to induce/repress RNA expression changes, and RNA changes were not associated with substantial changes of this histone mark in early AM immune response. On the other hand, DHMRs of H3K27ac were observed genome-wide, and the increase of H3K27ac at promoter regions of upregulated genes were correlated with expression levels of genes involved in inflammatory pathways such as TLR, NFκB, TNF or RIG-I like receptor signaling pathway. This is consistent with the activating role of this histone mark in gene expression and inflammatory response previously described (Denisenko et al., 2017), and similar results for H3K4me3 and H3K27ac patterns have been observed in mouse dendritic cells stimulated with LPS (Garber et al., 2012).

The less dramatic changes in chromatin states that we observed herein were primarily caused by dynamic changes of H3K27ac in AM due to stimulation with LPS or Poly (I:C) at 2h and 6h, especially in genes activated by TLR4, TLR3 and RIG-I receptor-mediated signaling. In spite of the H3K27ac changes in respond to treatments, the H3K27ac-DHMRs between timepoints did not show large changes at promoter regions of DEG. This could indicate that the induction of secondary responses did not require dramatic changes of H3K27ac from 2h to 6h, because response to stimuli from 2h and 6h are considered a transition between early and secondary transcriptional response in macrophages (Sharif et al., 2007). However, the enhancer of an early response gene, *TNF*, had changes in the chromatin state, switching from weak enhancer to strong enhancer 1kb upstream to the promoter region at 2h but not at 6h with respect to unstimulated controls. This change in the chromatin state was consistent with the increased expression of *TNF* at 2h after both LPS and Poly (I:C) stimulations and decreased expression at 6h LPS treatment and return to baseline for the 6h Poly (I:C) treatment. A similar pattern of H3K27ac modification near the promoter region of *TNF* have been observed in human nasopharyngeal epithelial cells in response to LPS: enrichment of binding sites of *RELA* and AP-1 members in H3K27ac peak regions in non-stimulated cells; and the increase of HM enrichment in the promoter region after LPS treatment, all contributing to the induction of the early response gene *TNF* (Borghini et al., 2018). In addition to the permissive chromatin for the immediate inflammatory response, we were able to identify an upregulated TF *NRF2*, which was associated with the increase of the H3K27ac motif enrichment in LPS and Poly(I:C) treatment. *NRF2* is a TF that suppresses macrophage LPS inflammatory response by blocking proinflammatory cytokine transcription through inhibiting RNA pol II recruitment (Kobayashi et al., 2016). Thus, our results indicate that although the regulatory regions of pro and anti-inflammatory genes are already open and in a poised state, an enhanced H3K27ac modification by 2h for *TNF* and 2-6h for *NRF2* was likely necessary to enhance the expression of the pro and anti-inflammatory response typically associated to macrophages response.

Taken together, the RNA-seq and ChIP-seq data suggests that the differential gene expression between non-stimulated and stimulated macrophages is determined at least partly by changes in chromatin accessibility to transcription factor motifs at active regulatory regions in the genome, and that these changes are mediated primarily through H3K27ac modifications. Although we found some individual genes that responded only for one treatment and were regulated at the epigenetic level, changes to chromatin states were relatively minor after stimulations at 2h and 6h using bacterial and viral mimics. This could suggest that regulatory elements (i.e., active promoters) are already active/poised and the chromatin is already open for immediate inflammatory response in porcine AM. In summary, our data reported here provides the first chromatin state of AM in response to bacterial and viral mimics, contributing to the Functional Annotation of Animal Genomes (FAANG) project (Giuffra et al., 2019). Furthermore, this work demonstrates an association of HMs, especially H3K27ac, with TFBM to mediate changes in gene expression in early macrophages responses to LPS and Poly (I:C).

## Data Availability Statement

Raw sequencing data from RNA-seq and ChIP-seq are available through the European Nucleotide Archive (project: PRJEB31483), https://www.ebi.ac.uk/ena/browser/view/PRJEB31483.

## Ethics Statement

The animal study was reviewed and approved by Institutional Animal Care and Use Committee – National Animal Disease Center (Ames, IA).

## Author Contributions

JH-U was responsible for collecting data for the whole study, and for drafting the manuscript. HL performed to the bioinformatic analysis and contributed to writing of the manuscript and the biological interpretation of the data. KB and ZB participated in the macrophage isolation, stimulation, and preservation for assays. CL performed the procedures for lung lavage collection of cells. CT and CL conceived and designed the project and participated in the interpretation and discussion of the results, as well as in the writing of the manuscript. All authors read and approved the final manuscript.

## Conflict of Interest

The authors declare that the research was conducted in the absence of any commercial or financial relationships that could be construed as a potential conflict of interest.

## Footnotes

1 http://www.bioinformatics.babraham.ac.uk/projects/fastqc/.

2 https://www.osti.gov/biblio/1241166-bbmap-fast-accurate-splice-aware-aligner

3 http://geneontology.org/

4 http://www.bioinformatics.babraham.ac.uk/projects/fastqc/

5 http://broadinstitute.github.io/picard/faq.html

6 https://gatk.broadinstitute.org/hc/en-us/articles/360035535932-Germline-short-variant-discovery-SNPs-Indels-

7 https://bioconductor.org/packages/release/bioc/html/DiffBind.html

8 https://stat.ethz.ch/R-manual/R-devel/library/stats/html/stats-package.html

9 https://cran.r-project.org/web/packages/ggfortify/index.html

10 https://www.animalgenome.org/repository/pub/ISU2020.0609/

## Abbreviations

*ACTB*: Beta-Actin
AM: Alveolar macrophages
*AMCF-II*: Alveolar macrophage-derived chemotactic factor-II
BH: Benjamini and Hochberg
ChIP: Chromatin immunoprecipitation
ChIP-seq: Chromatin immunoprecipitation and sequencing
CPM: Count per million reads mapped
DEG: Differential expressed gene
DHMRs: Differential histone modification regions
dsRNA: Viral double-stranded RNA
FAANG: Functional Annotation of Animal Genomes
FDR: False discovery rate
FRiP: Fraction of reads in peak regions
GO: Gene Ontology
h: Hours
H3K27ac: Histone H3 acetylated at lysine 27
H3K27me3: Histone H3 trimethylated at lysine 27
H3K4me1: Histone H3 monomethylated at lysine 4
H3K4me3: Histone H3 trimethylated at lysine 4
HCA: Hierarchical clustering analysis
HDACs: Histone deacetylases
HMDM: Human monocyte-derived macrophages
HMERs: Histone modification enriched regions
HMM: Hidden Markov Model
HMs: Histone modifications
IAV: Influenza A virus
IDR: Irreproducible discovery rates
IFN: Interferon
IGV: Integrative Genomics Viewer
KEGG: Kyoto Encyclopedia of Genes and Genomes
LPS: Lipopolysaccharide
MBMM: Mouse bone marrow-derived macrophages
MCODE: Molecular Complex Detection
Mhp: Mycoplasma hyopneumoniae
*MYOT*: Myotilin
PAMPs: Pathogen associated molecular patterns
PCA: Principal component analysis
PCV2: Porcine circovirus type 2
PMDM: Porcine monocytederived macrophages
Poly (I:C): Polyinosinic-polycytidylic acid
PRRs: Pattern recognition receptors
PRRSV: Porcine Reproductive and Respiratory Syndrome Virus
PTMs: Posttranscriptional modifications
QPCR: Real time polymerase chain reaction
RNA-seq: RNA sequencing
SLA: Swine leukocyte antigen
SVA: Surrogate variable analysis
TFBM: Transcription factor biding motif
TSS: Transcription start sites.
