# Supplementary material for: Changes in H3K27ac at Gene Regulatory Regions in Porcine Alveolar Macrophages Following LPS or PolyIC Exposure
Source: Front Genet. 2020 Aug 20;11:817. doi: 10.3389/fgene.2020.00817 (PMC7468443; doi:10.3389/fgene.2020.00817)
Supplement: Supplementary file 1 [file Presentation_1.pdf]

Supplementary figures

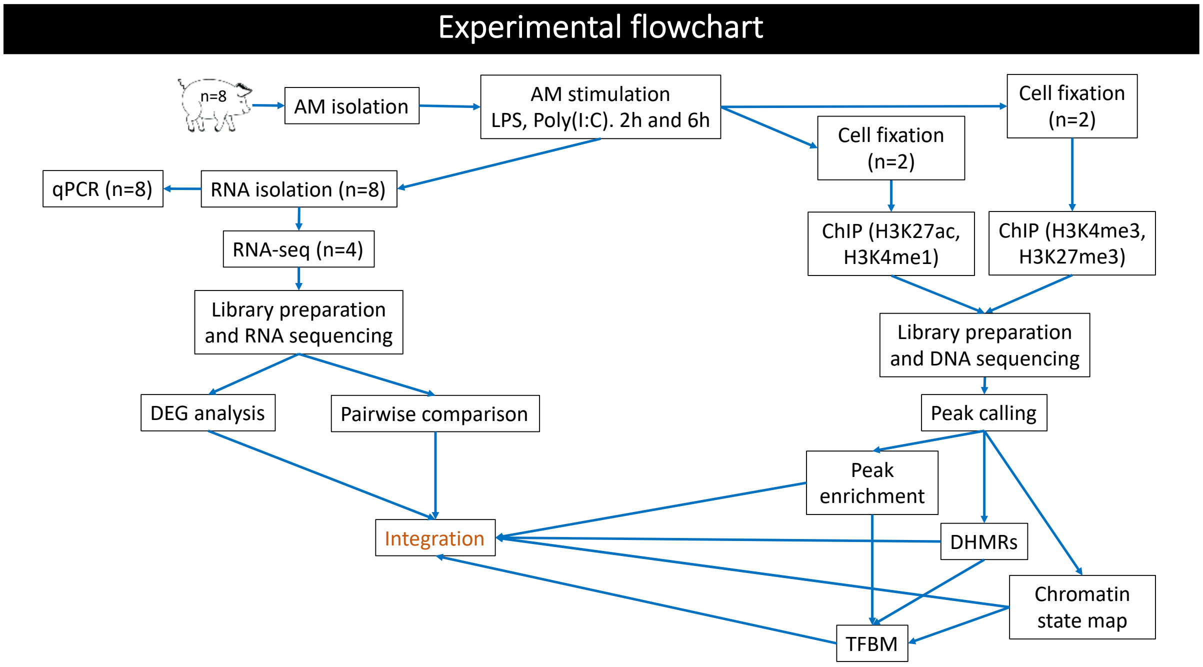

Supplementary figure S1. Experimental flowchart. AM: Alveolar macrophage; DEG: differentially expressed gene; DHMR: Differential histone modified region; TFBM: Transcription factor binding motif

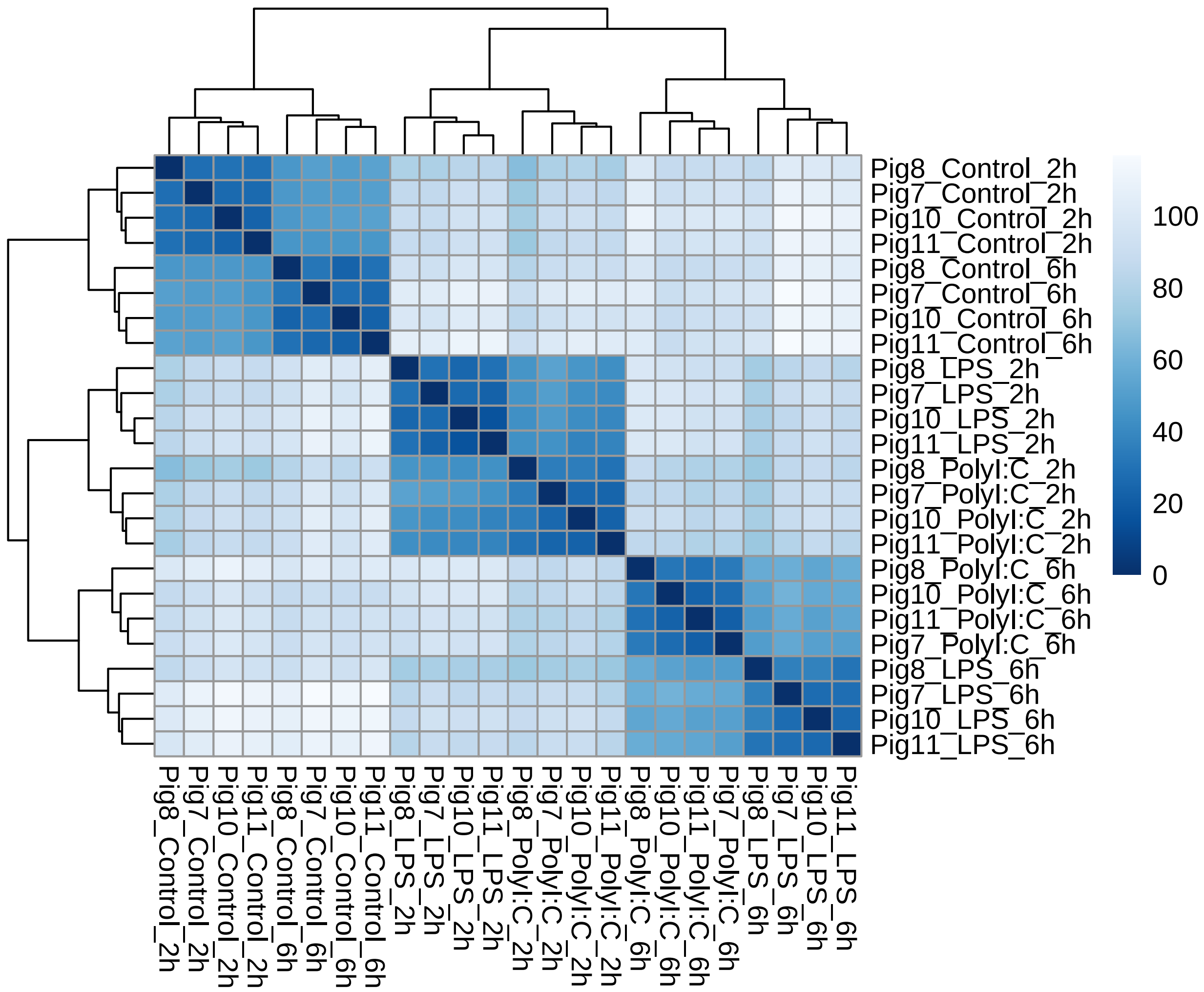

Supplementary figure S2. Transcriptional expression patterns of stimulated alveolar macrophages are distinct from non-stimulated cells and cluster more by time post-stimulation rather than treatment type. Heat map depicting hierarchical clustering of sample-to-sample distance. Gene expression for whole transcriptomes were used to calculate sample to sample Euclidean distance (color scale) for hierarchical clustering.

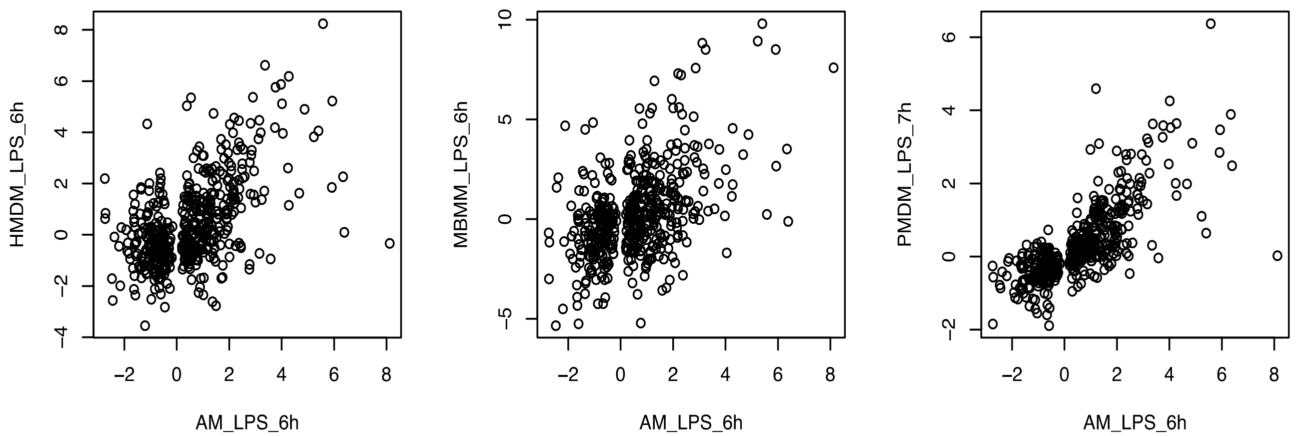

Supplementary figure S3. LPS-response genes show correlation across species. Spearman correlation of LPS-response genes in pig alveolar macrophages (AM) (6h) with human monocyte-derived macrophages (HMDM) (6h), mouse bone-marrow-derived macrophages (MBMM) (6h) and pig monocyte-derived macrophages (PMDM) (7h). As expected, the within-species comparison of PMDM with AM LPS-gene response data (R^2= 0.79, $P<2.2x10^{-16}$) was most correlated, followed by HMDM (R^2= 0.49, $P<2.2x10^{-16}$) and MBMM (R^2= 0.43, $P<2.2x10^{-16}$) responses relative to the AM data.

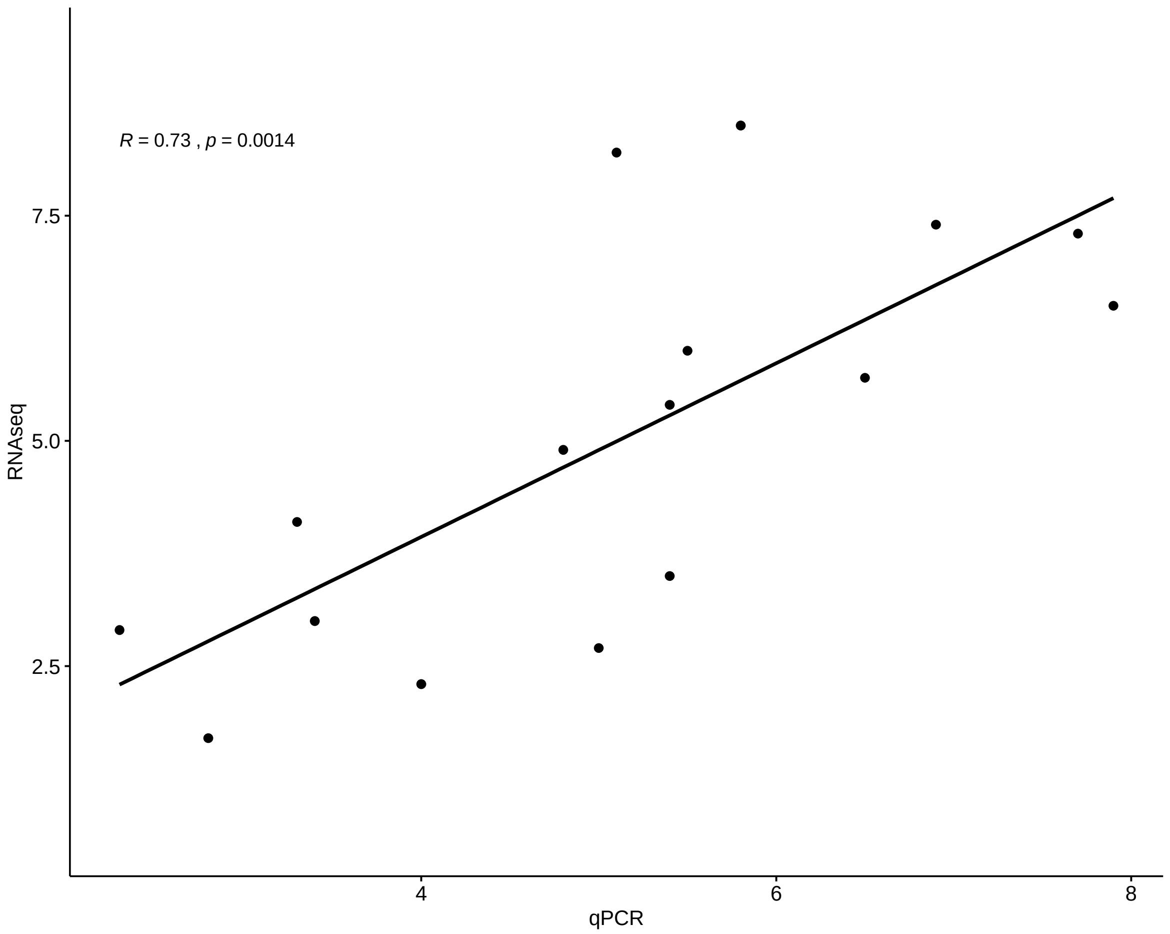

Supplementary figure S4. Inflammatory response gene validation by correlation between qPCR and RNA-seq. Pearson correlation analysis between RNA sequencing and quantitative real time PCR of differentially expressed inflammatory marker genes in alveolar macrophages stimulated with LPS and Poly(I:C) at 2h and 6h. Shaded regions depict 95% regression confidence intervals.

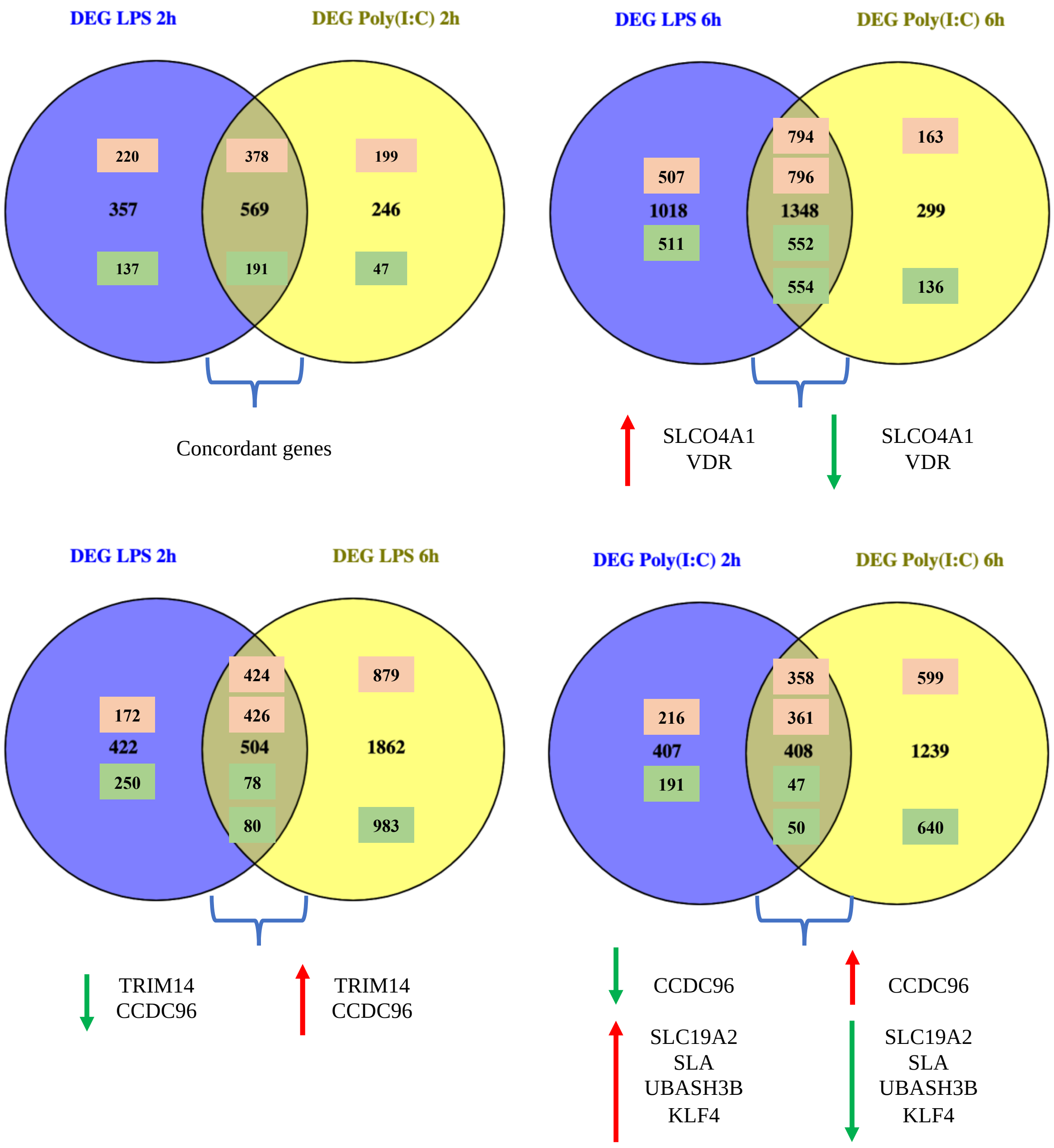

Supplementary figure S5. Distribution of number of DEG in alveolar macrophages among intersections between treatments and timepoints. Number of overexpressed genes are shown in red boxes and downregulated genes are shown in green boxes. Almost all common genes were concordant with the level of expression, except genes that were display under intersections. Common DEG genes are found in the center of the intersection and all DEG that response just for one treatment were display in the sides of the intersections. Genes which were DE in response to only one treatment were identified, and LPS treatment had a higher number of (unique) DEGs (LPS: 357-2h, 1018-6h; Poly (I:C): 246-2h, 299-6h). Common and unique DEG between treatments were annotated using GO terms. Common genes were enriched for similar biological functions (i.e. response to lipopolysaccharide, regulation of apoptotic process, cytokine-mediated signaling pathway etc.) and KEGG pathways (i.e. NF-kappa B signaling pathway, Toll-like

receptor signaling pathway, RIG-I-like receptor signaling pathway etc.) which we obtained before the treatments were analyzed independently. For unique DEGs, no additional enriched GO terms to those found in common DEGs between treatments were detected. However, at 6h the DEG detected following LPS treatment were enriched for KEGG pathways such as JAK-STAT signaling pathway (*CSF2, CSF3, IL10RA, IL12B, IL12RB1, IL23A, IL4R, LIF, SOCS3, SOCS7, STAT3, STAT5A, STAT6 etc.)* and chemokine signaling pathway *(AMCF-II, CCL17, CCL20, CCL22, CCL24, CCR1, CCR7, CXCL16, CXCR4, NFKBIB, STAT3*, etc.). Pathways noted following LPS treatment were different than pathways enriched in genes with a specific response to Poly (I:C) at 6h, which included KEGG pathways such as Th17 cell differentiation (*IL27RA*, *IL6R*, *IL6ST*, *PLCG1*, *RARA*, *SLA-DMB*, *SLA-DOA*, *SLA-DQB1*, *SLA-DRB1*) and antigen processing and presentation (*HSPA8*, *RFXAP*, *SLA-DMB*, *SLA-DOA*, *SLA-DQB1*, *SLA-DRB1*). The full list of enriched biological functions and KEGG pathways are available in Supplementary file S4. To determine the effect of the treatment time (2h and 6h) on the transcription response of AM to LPS and Poly (I:C), we compared the DEG lists (due to treatment) between timepoints within treatment (Supplementary file S4). After LPS treatment, a specific RNA response at 2h (422 DEG) was noted, including a set of genes that were DE at both 2h and 6h (504 DEG) and a large unique RNA response at 6h (1862 DEG). The direction of the response in genes that were DE at both times for LPS treatment were highly concordant between timepoints except for *TRIM14* and *CCDC96*, which were lower at 2h and higher at 6h (Supplementary Figure S3). In the LPS treatment time comparison, we did not identify specific GO terms enriched in RNA responses at 2h or 6h, except at 2h where DEG had enriched biological functions related to the negative regulation of transcriptional response, and at 6h where DEG had enrichment for the bile secretion pathway. After Poly (I:C) treatment unique RNA responses at 2h (407 DEG) were noted, and a set of genes DE at both 2h and 6h (508 DEG). Genes DE at both timepoints were highly concordant, except for *CCDC96* (lower at 2h and higher at 6h), *SLC19A2*, *SLA*, *UBASH3B* and *KLF4* (higher at 2h and lower at 6h). As noted for the LPS treatment, DEG following Poly (I:C) treatment at 2h and 6h had similar enriched biological functions and KEGG pathways, although “proteasomal ubiquitin-independent protein catabolic process” (NFE2L2, *PSMA2*, *PSMA3*, *PSMA5*, *PSMA6*, *PSMB8*, *PSMB9*) was enriched only at 6h.

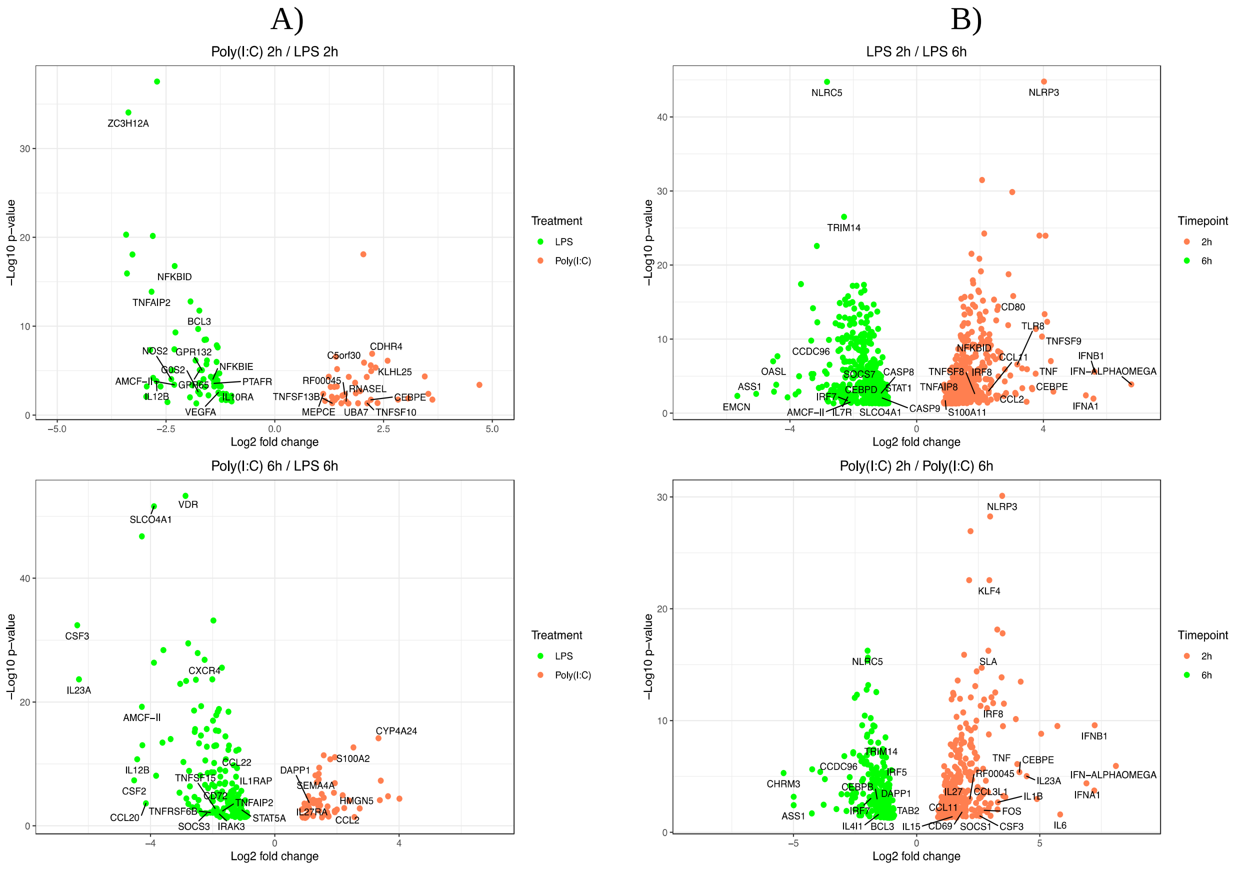

Supplementary figure S6. Direct pairwise comparison of gene expression among treatments and times post-treatments. On the left between Poly (I:C) and LPS, where green dots show higher expression in LPS, and orange dots show higher expression in Poly(I:C) at 2 hours (top) and 6 hours(bottom). On the right, pairwise comparison of gene expression between timepoints 2h (orange dots) and 6h (green dots) for LPS (top) and Poly (I:C) (bottom). Non-stimulated AM data at 2h and 6h were subtracted it out to perform the pairwise comparison. Volcano plot showing highly differentially expressed genes in each treatment at 2h and 6h (genes with $|log_2(FC)| > log_2(1.5)$ and adjusted p-value < 0.05 were displayed). Genes involved in related inflammatory GO term were labeled. The pairwise analysis between treatments revealed many genes have higher expression level in response to LPS (61 genes, 2h; 179 genes, 6h) than Poly (I:C) (42, 2h; 62, 6h) stimulation at both timepoints (Supplementary file S5).

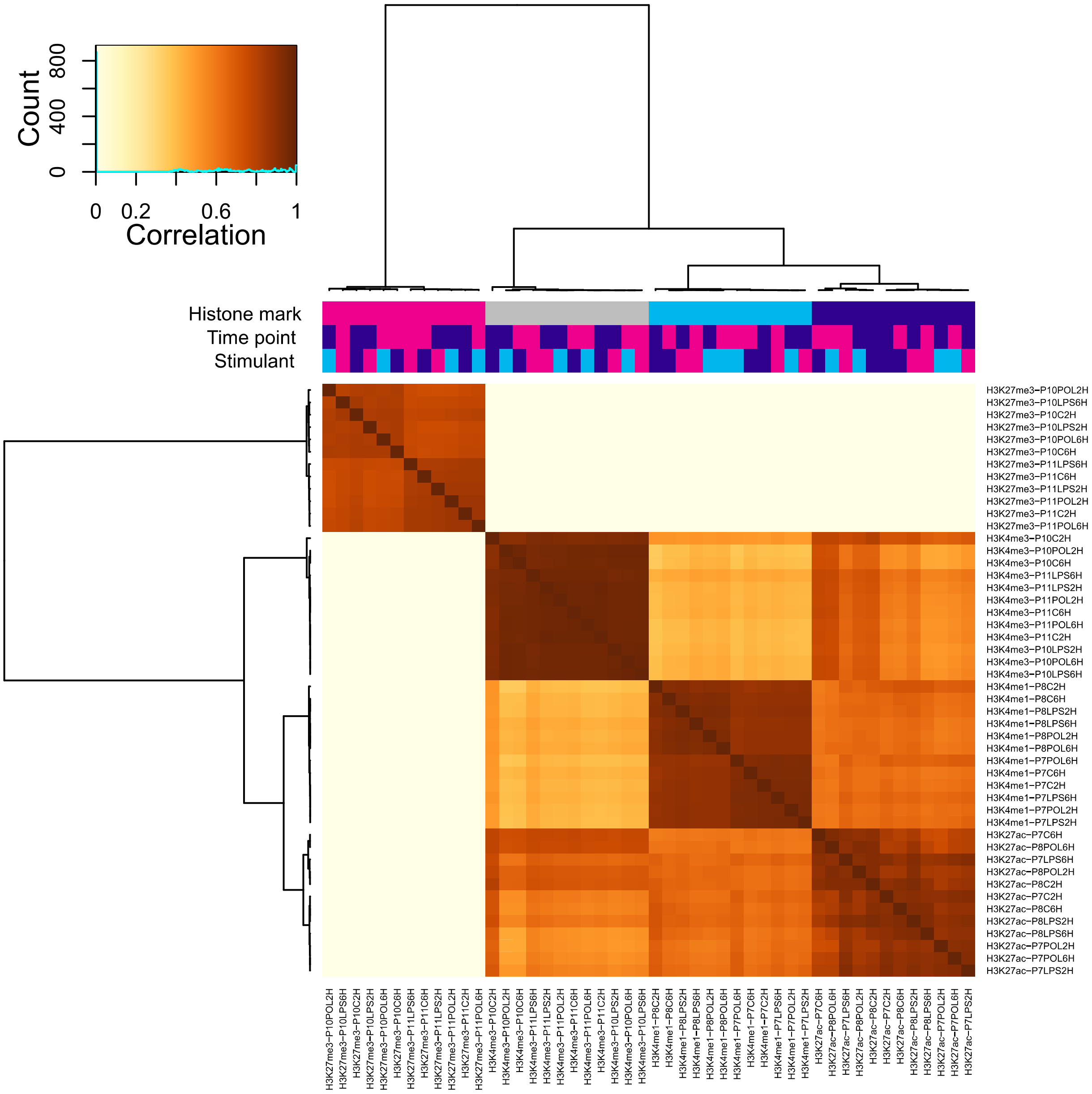

Supplementary figure S7. Correlation heatmap of histone ChIP-seq. Heat map displaying Spearman rank correlations between all ChIP-seq samples. Spearman correlations were calculated using the normalized read depth across the entire set of chromatin modification sites identified for all ChIP-seq experiments.

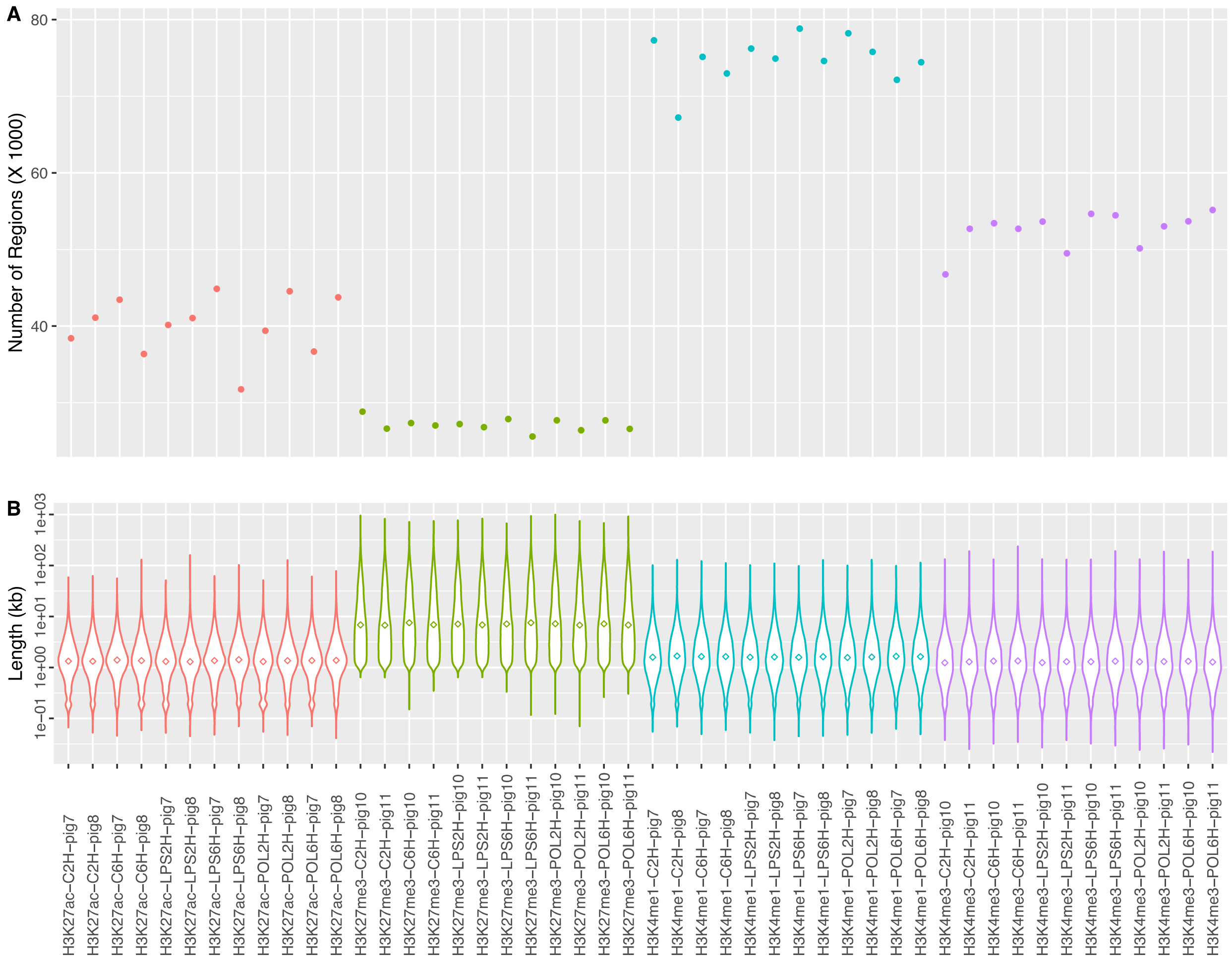

Supplementary figure S8. Histone modification peaks. (A) Distribution of the number of peaks called over AM samples and treatments. (B) Distribution of the peak length (Kb) of individual AM samples. The diamond inside the plot represents the median.

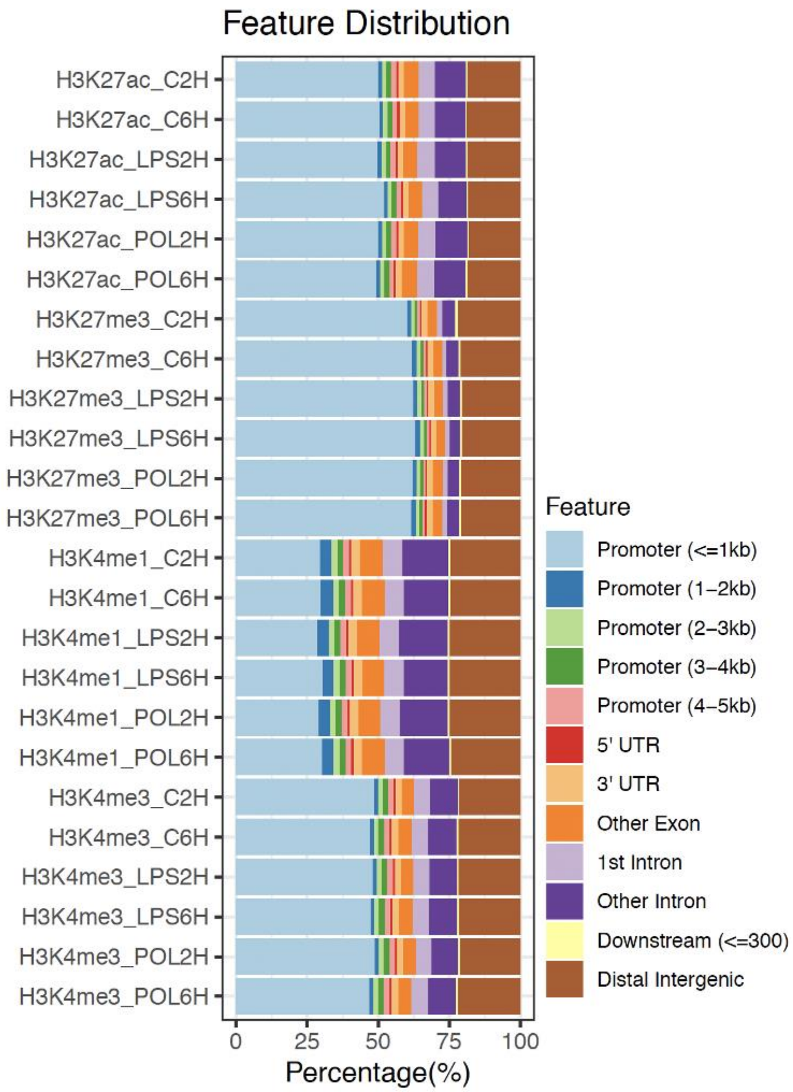

Supplementary figure S9. ChIP-seq peak annotation distribution on the porcine genome. Controls, LPS and Poly (I:C) treatments were labeled as C, LPS and POL respectively). 2H: 2 hours post treatment; 6H: 6 hours post-treatment.

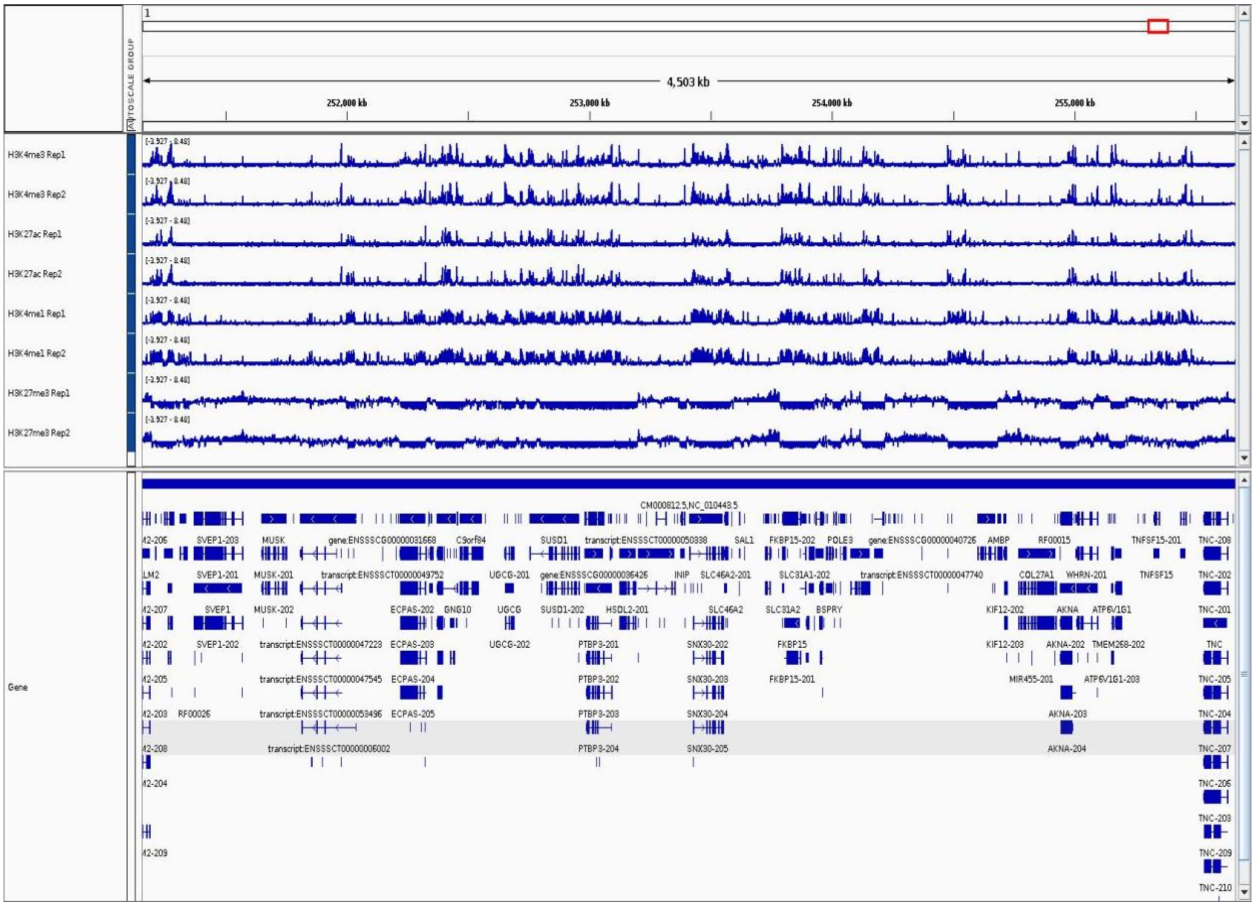

Supplementary figure S10. IGV screenshot of H3K4me, H3K27ac, H3Kme1 and H3K27me3 replicates across the porcine genome annotation (*Sus scrofa* 11.1, Ensembl, version 90) shows good consistency between replicates.

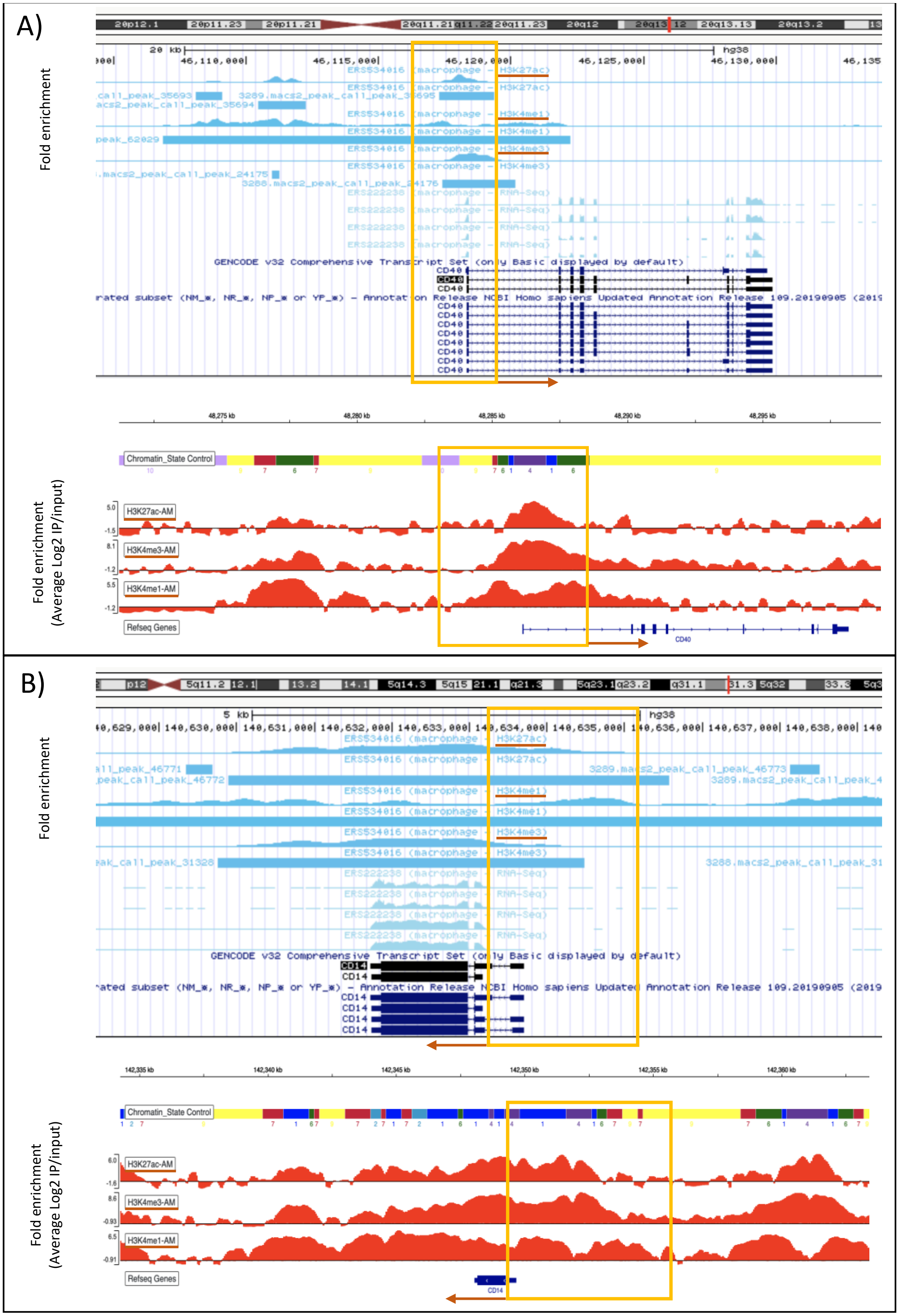
A)
Fold enrichment
Fold enrichment
(Average Log2 IP/input)
GENCODE V32 Comprehensive Transcript Set (only Basic displayed by default)
CD40
Chromatin_State Control
H3K27ac-AM
H3K4me3-AM
H3K4me1-AM
Refseq Genes
B)
Fold enrichment
Fold enrichment
(Average Log2 IP/input)
GENCODE V32 Comprehensive Transcript Set (only Basic displayed by default)
CD14
Chromatin_State Control
H3K27ac-AM
H3K4me3-AM
H3K4me1-AM
Refseq Genes

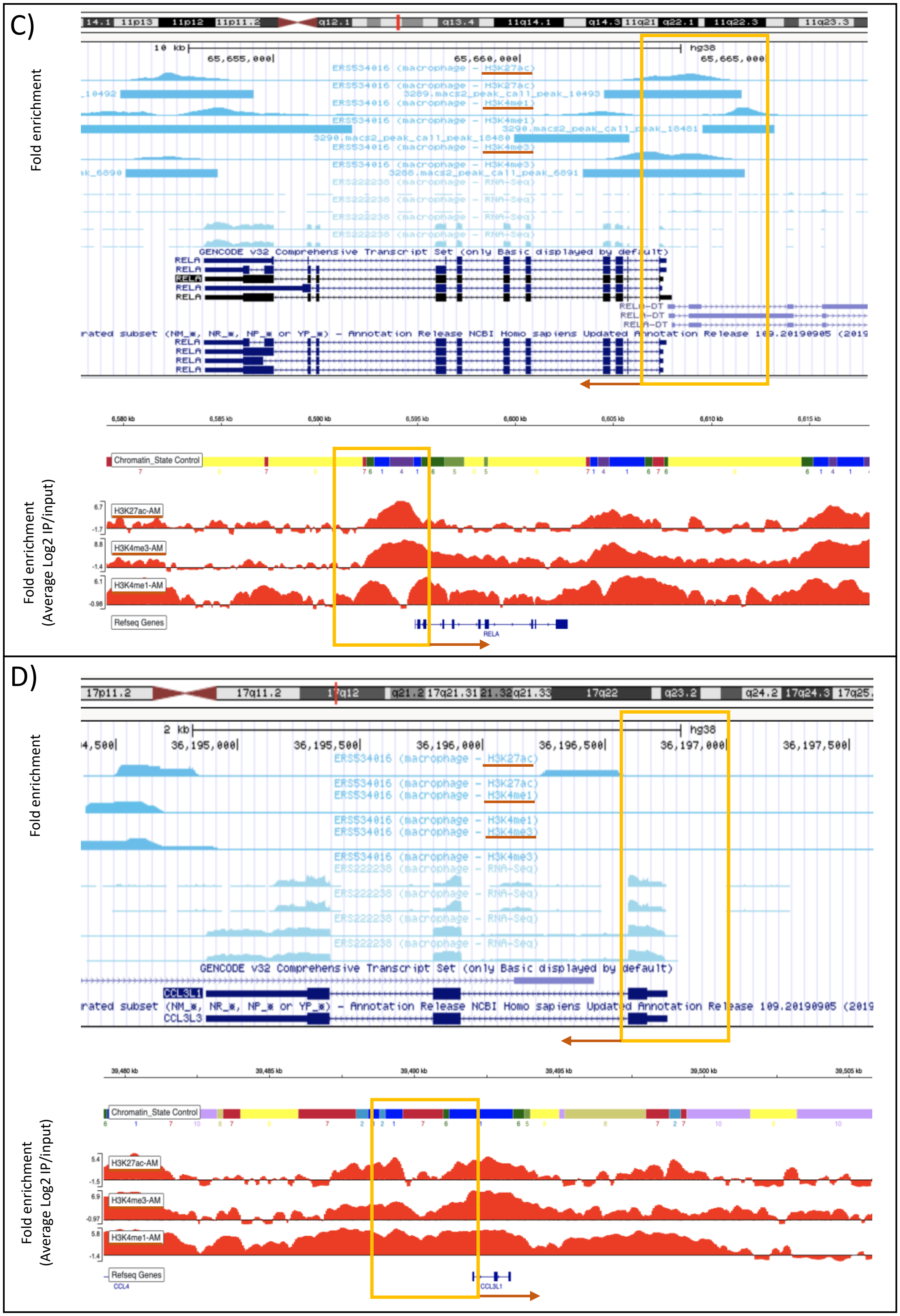
C)
Fold enrichment
GENCODE v32 Comprehensive Transcript Set (only Basic displayed by default)
RELA
RELA-DT
hg38
10 kb
65,655,000
65,660,000
65,665,000
ERS534016 (macrophage - H3K27ac)
ERS534016 (macrophage - H3K4me1)
ERS534016 (macrophage - H3K4me3)
ERS222238 (macrophage - RNA-Seq)
Fold enrichment
(Average Log2 IP/input)
Chromatin_State Control
H3K27ac-AM
H3K4me3-AM
H3K4me1-AM
Refseq Genes
6,580 kb
6,585 kb
6,590 kb
6,595 kb
6,600 kb
6,605 kb
6,610 kb
6,615 kb
D)
Fold enrichment
GENCODE v32 Comprehensive Transcript Set (only Basic displayed by default)
CCL3L1
CCL3L3
hg38
2 kb
36,195,000
36,195,500
36,196,000
36,196,500
36,197,000
36,197,500
ERS534016 (macrophage - H3K27ac)
ERS534016 (macrophage - H3K4me1)
ERS534016 (macrophage - H3K4me3)
ERS222238 (macrophage - RNA-Seq)
Fold enrichment
(Average Log2 IP/input)
Chromatin_State Control
H3K27ac-AM
H3K4me3-AM
H3K4me1-AM
Refseq Genes
CCL4
CCL3L1
39,480 kb
39,485 kb
39,490 kb
39,495 kb
39,500 kb
39,505 kb

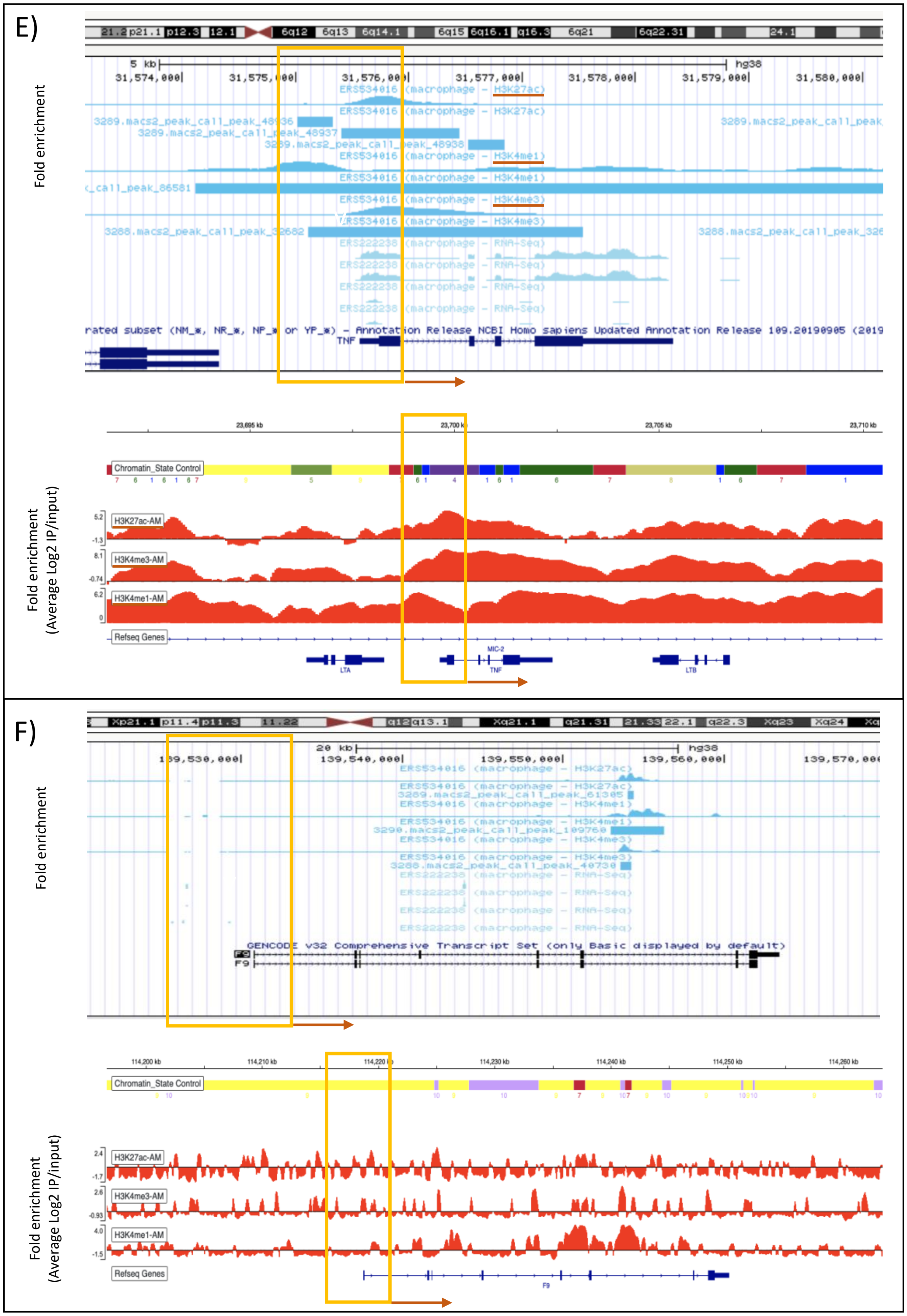

E)
Fold enrichment
5 kb
hg38
31,574,000
31,575,000
31,576,000
31,577,000
31,578,000
31,579,000
31,580,000
ERS534016 (macrophage - H3K27ac)
ERS534016 (macrophage - H3K4me1)
ERS534016 (macrophage - H3K4me3)
ERS222238 (macrophage - RNA-Seq)
TNF
Fold enrichment (Average Log2 IP/input)
Chromatin_State Control
H3K27ac-AM
H3K4me3-AM
H3K4me1-AM
Refseq Genes
LTA
MIC-2
TNF
LTB
F)
Fold enrichment
20 kb
hg38
139,540,000
139,550,000
139,560,000
ERS534016 (macrophage - H3K27ac)
ERS534016 (macrophage - H3K4me1)
ERS534016 (macrophage - H3K4me3)
ERS222238 (macrophage - RNA-Seq)
GENCODE v32 Comprehensive Transcript Set (only Basic displayed by default)
F9
Fold enrichment (Average Log2 IP/input)
Chromatin_State Control
H3K27ac-AM
H3K4me3-AM
H3K4me1-AM
Refseq Genes
F9

Supplementary figure S11. Selected epigenetic mark comparisons for human and pig genes. UCSC (for human macrophages, Human GRCh38.p13) and IGV (for porcine AM, *Sus scrofa* 11.1, Ensembl, version 90) screenshots of H3K4me, H3K27ac and H3Kme1 across the gene body of macrophage-expressed genes involved in TLR signaling pathways (A) *CD40*, (B) *CD14*, (C) *RELA*, (D) *CCL3L1*, (E) *TNF* , and a non-expressed gene in macrophages (*F9*).

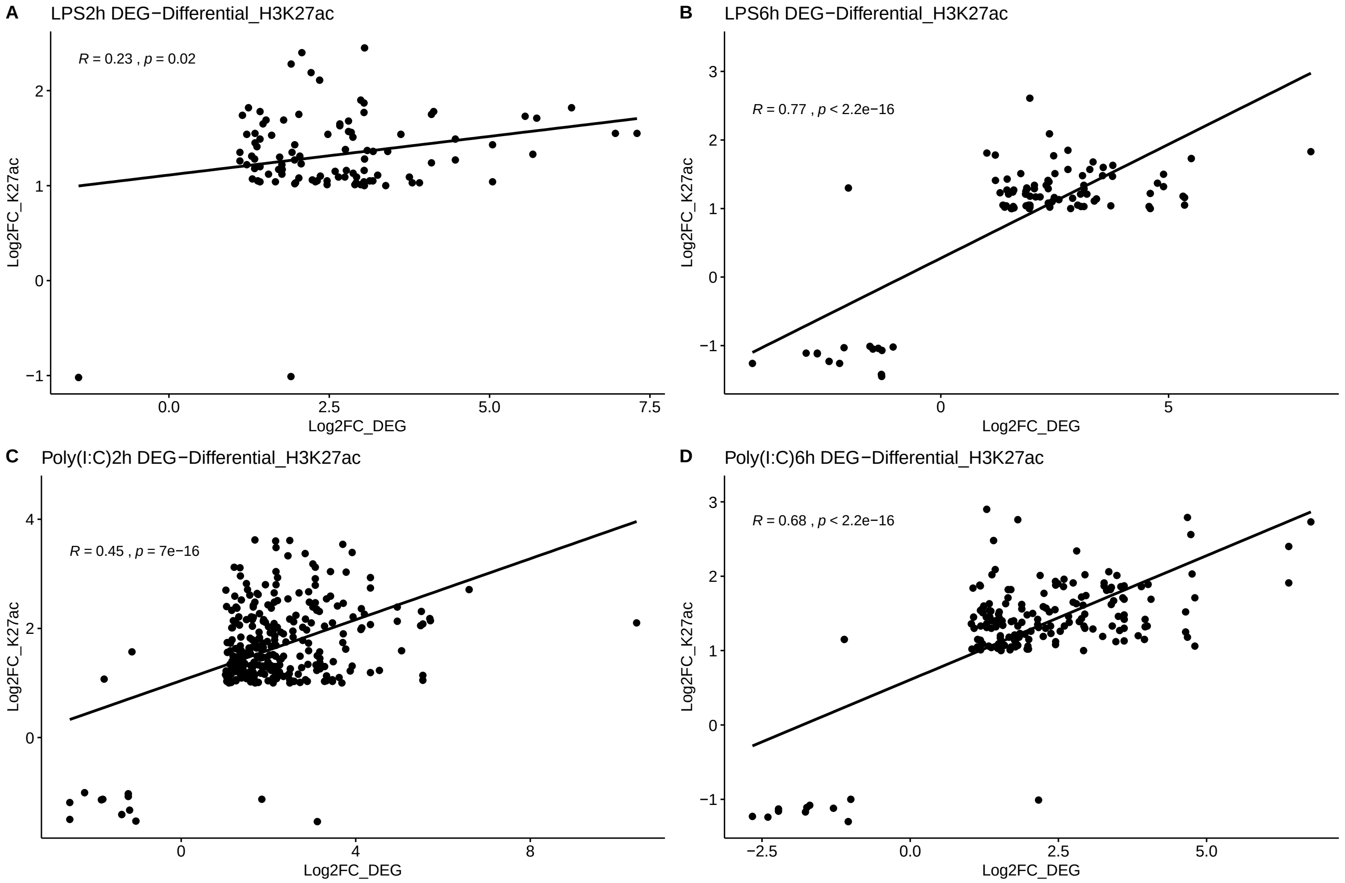

Supplementary figure S12. Correlation between changes in H3K27ac LPS-response genes with changes in expression due to treatment for differentially expressed genes (DEG). For each H3K27ac differential peak (y axis) near to the promoter region (5Kb), the Log2 fold change of the DEG for LPS response (x axis) were plotted. Correlation between these two data sets was tested with a Pearson test. Significance was set at $P<0.05$. Shaded regions depict 95% regression confidence intervals.

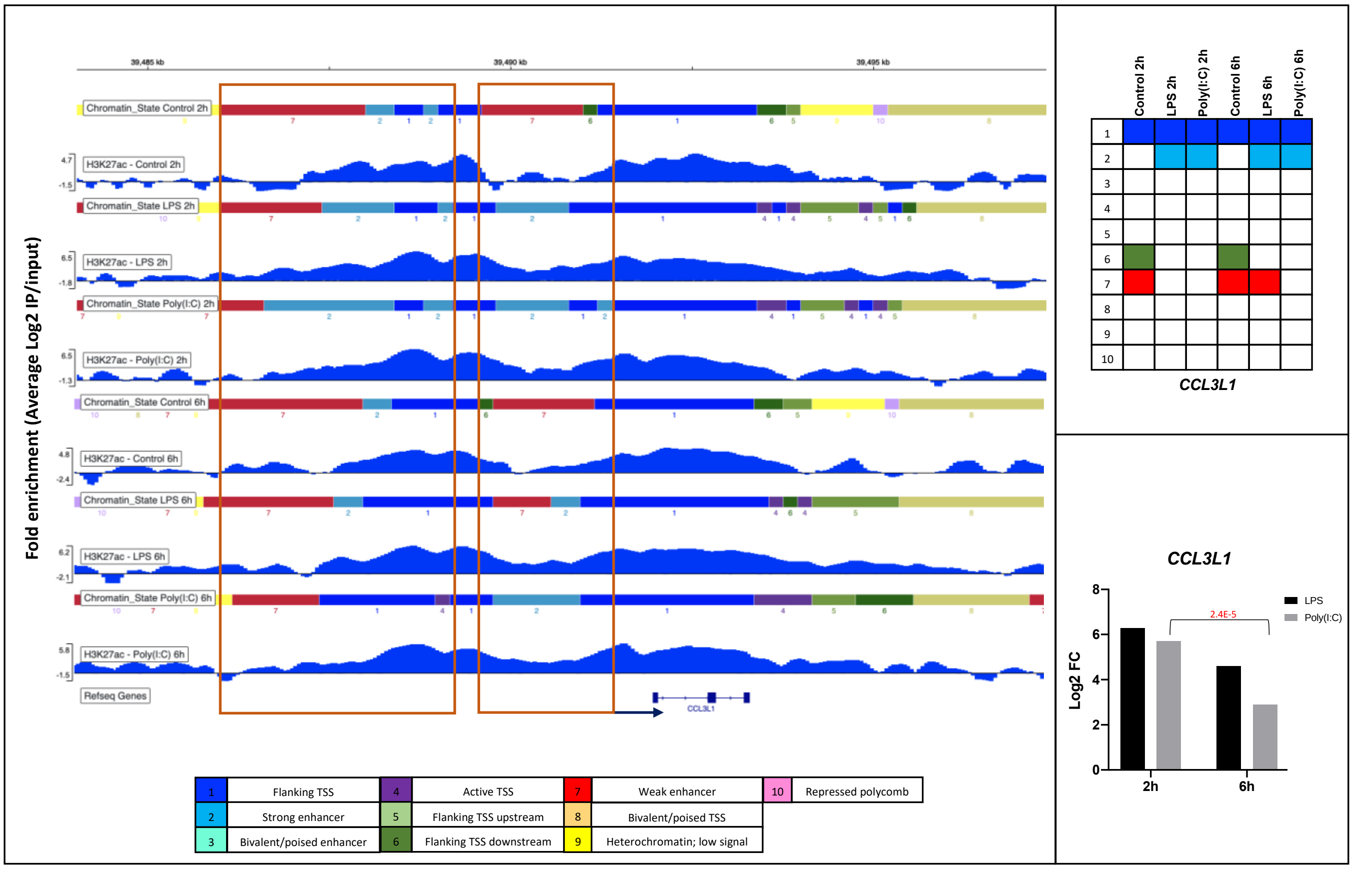

Supplementary figure S13. Changes of chromatin state of *CCL3L1* gene in response to LPS and Poly(I:C) at 2h and 6h. A) IGV screenshots showing DHMRs-H3K27ac with chromatin states around 1kb of promoter regions of *CCL3L1* gene in respond to treatments. Annotation of the chromatin states is shown as legend below figure and as a (B) summary table. C) Gene expression values of *CCL3L1* gene from RNA-seq of stimulated AM shows association of gene expression changes with chromatin state changes.
